# The Relation Between Attachment and Depression in Children and Adolescents: A Multilevel Meta-Analysis

**DOI:** 10.1007/s10567-019-00299-9

**Published:** 2019-08-07

**Authors:** Anouk Spruit, Linda Goos, Nikki Weenink, Roos Rodenburg, Helen Niemeyer, Geert Jan Stams, Cristina Colonnesi

**Affiliations:** 1 Research Institute of Child Development and Education, Faculty of Social and Behavioural Sciences, University of Amsterdam, Nieuwe Achtergracht 127, PO box 15776, 1001 NG Amsterdam, The Netherlands; 2 Stichting Philadelphia Zorg, Amersfoort, The Netherlands; 3 Kristal, Centrum voor Verstandelijke Beperking en Psychiatrie, GGZ Rivierduinen, Leiden, The Netherlands; 4 Stichting Epilepsie Instellingen Nederland, Heemstede, The Netherlands; 5 Clinical-Psychological Intervention, Department of Education and Psychology, Freie Universität Berlin, Berlin, Germany

**Keywords:** Attachment, Depression, Children, Adolescents, Multilevel meta-analysis

## Abstract

Insecure attachment to primary caregivers is associated with the development of depression symptoms in children and youth. This association has been shown by individual studies testing the relation between attachment and depression and by meta-analyses focusing on broad internalizing problems instead of depression or adult samples only. We therefore meta-analytically examined the associations between attachment security and depression in children and adolescents, using a multilevel approach. In total, 643 effect sizes were extracted from 123 independent samples. A significant moderate overall effect size was found (*r* = .31), indicating that insecure attachment to primary caregivers is associated with depression. Multivariate analysis of the significant moderators that impacted on the strength of the association between attachment security and depression showed that country of the study, study design, gender, the type of attachment, and the type of instrument to assess attachment uniquely contributed to the explanation of variance. This study suggests that insecure attachment may be a predictor of the development of depression in children and adolescents. When treating depression in children, attachment should therefore be addressed.

**Electronic supplementary material:**

The online version of this article (10.1007/s10567-019-00299-9) contains supplementary material, which is available to authorized users.

## Introduction

From early infancy on, children seek the proximity of a sensitive and responsive caregiver for protection and security (Bowlby 1969, 1988). Early relationship experiences with caregivers lead to generalized expectations about the self, the world, and others. On the basis of these experiences, children develop an internal working model of significant relationships with others. If the caregiver is consistently sensitive to the child’s proximity seeking behavior, the child will be securely attached and will perceive the caregiver as a safe haven and as a secure base from which the environment can be explored (Bowlby 1969, 1988). When caregivers are inconsistently sensitive, show a lack of sensitivity or are even frightening to the child, children are at risk for developing insecure attachment relationships (Ainsworth et al. 1978; Main and Solomon 1990).

Previous (meta-analytic) studies have shown that insecurely attached children are at greater risk for psychopathology, such as internalizing and externalizing problems (Colonnesi et al. 2011; Groh et al. 2017; Hoeve et al. 2012; Madigan et al. 2016). Although several studies investigated the relation between attachment and internalizing symptoms, the strength of the specific relation between attachment security and depression symptoms or disorder has not yet been meta-analytically investigated. Studying the association between attachment and depression is important in order to understand whether attachment insecurity and depression co-occur, and to learn about the predictive value of attachment for depression in youth. Therefore, in the current study, we investigate the relation between attachment and depression in children and adolescents by means of a three-level meta-analysis, an approach to meta-analysis facilitating the examination of both within and between-study heterogeneity (Van den Noortgate and Onghena 2003).

### Secure and Insecure Attachment Patterns

Attachment theory has been developed by John Bowlby (1969, 1988). Research into child attachment took flight when a laboratory procedure was devised: the Strange Situation Procedure (Ainsworth et al. 1978). This procedure was meant to assess the nature of the child’s attachment to the main caregiver, and to provide an empirical base to attachment theory. Through this procedure, the child can be classified as secure (Type B), insecure avoidant (Type A), and insecure defensive or insecure ambivalent (Type C). Children with a secure attachment have a good balance between the urge to explore the environment and their attachment behaviors, that is, seeking proximity to the caregiver (Ainsworth et al. 1978). Children with an insecure-avoidant attachment pattern minimize their attachment behaviors as they have experienced rejection or consistent low sensitivity from their caregiver(s). Insecure resistant or ambivalent attached children have experienced inconsistent sensitivity from their caregiver, and maximize attachment behaviors in order to preserve attention from the caregiver (Ainsworth et al. 1978).

Later, it was found that some children could not be categorized according to the three insecure attachment categories. A fourth category of attachment was, therefore, added: disorganized/disoriented attachment (Type D; Main and Solomon 1990). Disorganized attached children perceive their caregivers as a source of both comfort and fear, which is an unsolvable paradox. This fear with no solution prohibits the development of an organized strategy for the use of the attachment figure in case of distress and results in a mixture of different types of insecure behaviors combined with frightening reactions towards the caregiver (Main and Hesse 1990). Children of parents with severe psychopathology and maltreated children often show these disorganized attachment behaviors (Radke-Yarrow et al. 1985; Van IJzendoorn et al. 1999). Thus, attachment relationships can be classified as secure, insecure-avoidant, insecure-ambivalent, and insecure-disorganized attachment.

The distribution of the specific types of attachment relationships has been meta-analytically examined by Van IJzendoorn et al. (1999) in the general population, parents of low SES and a number of clinical groups. In the general population, 62% of the young children were securely attached to their primary caregiver, whereas the insecure attachment patterns were less frequently diagnosed in parent–child relationships: insecure avoidant (15%), insecure ambivalent (9%), and disorganized (15%). Notably, in maltreated samples, only 9% of the children were securely attached to their parents, 28% avoidantly attached, 15% ambivalently attached, and a large percentage of 48% of the children were classified as disorganized (Van IJzendoorn et al. 1999).

### Depression in Youth

Depression is a serious mood disorder that affects children’s and adolescents’ physical, emotional, and social-cognitive development, and it is characterized by feelings of sadness and lack of interest or pleasure (Clark et al. 2012). Depression disorder is diagnosed in children and adolescents since the 1970’s (Schulterbrandt and Raskin 1977). The main symptoms are depressed mood, irritability, feelings of worthlessness and guilt, problems with sleeping, weight loss, thoughts of death and suicide, or withdrawal from (social) activities. The symptoms can vary from mild to severe, and they must last at least 2 weeks for a diagnosis of depression. In addition, the DSM-5 contributed to better recognition and understanding of depression in childhood, adding two crucial distinctions between symptoms in children and adults. First, children may express irritability rather than sad or depressed mood. Second, weight loss may result in failure to reach appropriate weight milestones (DSM-5; American Psychiatric Association 2013). Depression symptoms can be present also before or without a diagnosis of depression. Antecedents of children’s and adolescents’ symptoms of depression are investigated in the general and clinical population as well as the possible developmental consequences of depression.

The prevalence rate of a childhood diagnosis of depression is around 3% before the age of 13 years, and 6% between 13 and 18 years, with greater increases among females than males (Costello et al. 2006). Risk factors can be categorized as biologic (e.g., depression in the family, sex, hormonal changes during puberty, sleeping or eating problems), psychologic (e.g., insecure or disorganized attachment, self-consciousness, negative thinking style or self-evaluation), or environmental (e.g., trauma, parental conflict, poor sibling or peer relationships, low SES; Brenning et al. 2011; Clark et al. 2012). About 40–70% of depressed children and adolescents present at least one other disorder, mainly related to anxiety or other internalizing problems (Rohde 2009). Consequences of depressions are of great importance for the individual socio-emotional development of children and youth. Consequences of depression are suicide behavior, substance abuse, poor academic and work performance, and poor relationships with family and friends (Fergusson and Woodward 2002; Keenan-Miller et al. 2007). It is, therefore, important to get insight into the risk factors for depression symptoms in order to better predict them, providing prospects for preventive interventions.

### The Relation Between Attachment and Depression

Secure attachment is a protective mechanism that prevents the development of psychopathology, while insecure attachment is correlated with child psychopathology, in particular, internalizing problems, including depression (Colonnesi et al. 2011; Groh et al. 2012; Madigan et al. 2013). To understand why an association between attachment and depression is expected and to show the relevance of examining this association, we describe four theories that explain this association.

A first explanation for the association between attachment security and depression is that the quality of the early attachment experiences with caregivers shape the internal working model of the child. This is a set of generalized relationship expectancies about the self and others (Bowlby 1969). This cognitive-affective schema organizes the identification, interpretation, categorization, and evaluation of (attachment-related) experiences (Bosmans et al. 2010; Dozois and Beck 2008; James et al. 2007). Repeated experiences of unavailability of the caregiver could lead to dysfunctional cognitions (i.e., cognitive schemas) about the self (*I am worthless*) and others (*Nobody cares about me*, *Others are not available for me*), which may enhance the risk for depressive symptoms (Dozois and Beck 2008). Dysfunctional schemas are one of the core elements of Beck’s (1967) cognitive model of depression, and can, therefore, mediate the relation between insecure attachment and symptoms of depression. From this perspective, it is not surprising that especially the cognitive schemas of expectations to be rejected or disconnected mediated the association between attachment insecurity and psychopathology (Bosmans et al. 2010). For a full discussion on the integration of the cognitive schema theory and internal working models, we refer to Bosmans (2009).

A second explanation comes from research on chronic stress. One of the core assumptions of Bowlby’s attachment theory (1969) is that infants and young children need their caregiver(s) for stress and emotion regulation. The more the caregiver is able to help the child in reducing distress and comfort, the more secure the attachment relationship will be. Caregivers of insecurely attached children generally have more difficulties to comfort their child compared to caregivers of securely attached children, for example, because of their own negative attachment experiences or other mental health issues. Children need the comfort of their caregivers in order to learn self-regulation (Evans and Porter 2009). The absence of comfort may result in elevated stress levels throughout their childhood, because the parents are not able to successfully guide their children through the stressful events that are part of typical development (Brenning et al. 2012; Cassidy 1994). Studies have repeatedly shown the significance of chronic stress in depression, mostly explained by neuro(psycho)logical mechanisms (Banasr et al. 2017; Juster et al. 2010). Moreover, secure attachment relationships with caregivers predict the quality of social relationships later in life. Meaningful, supportive social relationships have been shown to buffer the effects of stress on depression (Jaremka et al. 2013).

Third, the co-occurrence of attachment problems and depression could be explained by genetic vulnerability of depression. Genetic markers have been associated with depression symptoms and disorders (Hyde et al. 2016; Wray et al. 2018). At the same time, depressive symptoms in mothers have been identified as an important predictor of insecure attachment relationships with their child (Graffi et al. 2016). The genetic vulnerability of depressed mothers could be transferred to their children, resulting in an increased risk for depression in their children, while the depressive symptoms in mothers lead to insecure attachment relationships with their child.

Finally, the co-occurrence of attachment problems and depression can be explained by the transfer of social-ecological risk factors from caregivers to children, including low socioeconomic status, debts, migration status, inadequate housing, general health, and low quality of the supportive system around the family. Numerous studies showed the intergenerational transmission of social-ecological context (Black et al. 2005; Coneus and Spiess 2012; Sharkey 2008). Social-ecological risk factors have been both predictive of attachment insecurity and depression in youth (Cyr et al. 2010; Hopkins et al. 2013; Raikes and Thompson 2005) contributing to the explanation of the association between attachment security and depression.

Thus, previous research indicates that there are several indications that attachment is a predictive factor of depression symptoms in youth and that attachment insecurity and depression co-occur. However, empirical evidence for this relation comes mainly from individual studies testing the relation between attachment and depression, from meta-analyses on broad internalizing problems (e.g., Groh et al. 2012; Madigan et al. 2016) or a meta-analysis on adult samples (Dagan et al. 2018).

We believe it is important to study the association with attachment for specific types of internalizing problems. Brumariu and Kerns (2010) found in a narrative review more consistent associations between attachment and depression or anxiety than between attachment and internalizing symptoms assessed at a global level. The latest meta-analyses on the association between attachment and internalizing problems showed inconsistent results: *r *=.07 (Groh et al. 2012), *r *= .18 (Madigan et al. 2013), and *r *= .28 (Madigan et al. 2016). The meta-analytic study of Colonnesi et al. (2011), with an effect size of *r *= .30 for the relation between attachment and anxiety, indeed suggests that the association between attachment and psychopathology is larger for specific anxiety or depression alone than for the broad-band category of internalizing problems. Further, although the internalizing disorders and symptoms are correlated and comorbid, these are classified as separate disorders, with distinct characteristics. Understanding the different correlates and risk factors of depression compared to other internalizing problems could improve the available interventions for specific internalizing problems. To conclude, a meta-analysis on the relation between attachment and depression that accounts for both within and between-study differences could provide a more in-depth investigation of this association and its potential moderators, with important implications for research and clinical practice.

### The Current Study

We sought to investigate the association between attachment and depression in youth and to examine which moderators influence the relation between attachment and depression. We included both cross-sectional and longitudinal studies in the current meta-analysis to be able to examine whether attachment insecurity and depressive symptoms co-occur and to test whether attachment insecurity is a risk factor for depressive symptoms. The current meta-analytic study applies a multilevel approach, which allows for comprehensive moderator analyses to assess the influence of study, sample, attachment, and depression characteristics on the relation between attachment and depression (Van den Noortgate and Onghena 2003).

Previous studies have shown various moderators of the relation between attachment and internalizing problems (Colonnesi et al. 2011; Groh et al. 2012; Madigan et al. 2013), and it is interesting to assess if similar moderating effects are present for studies with depression as dependent variable. The specific moderators of the current study are described and justified in the Methods section. The multilevel meta-analytic techniques enable the use of all available effect sizes in the analyses, so all information can be preserved and maximum statistical power is generated (Assink et al. 2015).

In sum, the first aim of the current multilevel meta-analysis is to investigate the strength of the relation between attachment security and depression in children and adolescents. Second, it aims to examine which moderators influence the relation between attachment and depression.

## Method

### Inclusion Criteria

All studies available from 1978 until June 2017 addressing the relation between attachment and depression from infancy to adolescence were included in the current meta-analysis. Multiple inclusion criteria were formulated to select the studies for the present review. First, the studies had to include a measure of the child’s attachment and of child’s depression. Second, studies with samples with an age range between 0 and 23 years were included. Third, only studies reporting on children’s attachment to their parents and/or primary caregivers were included in the present meta-analysis. Fourth, only studies in English, German, French, Italian, Portuguese, or Dutch were included. Fifth, the studies had to provide sufficient statistical information to calculate an effect size. Finally, only studies reporting on bivariate associations between attachment and depression were included, because in multivariate effect sizes, the set of covariates varies greatly among different studies. Therefore, combining and comparing differently adjusted effect sizes limit the ability to estimate a true overall relation between attachment and depression (Mulder et al. 2018).

We excluded studies on the relation between the child’s attachment relationship and maternal depression or studies reporting on associations between the child’s attachment to other persons (such as peers). Furthermore, studies reporting on internalizing symptoms but not specifically on depression (e.g., using the internalizing, anxious/depressed or social withdraw scales of the CBCL/YSR) and studies on parental bonding, family cohesion, or family conflict were excluded.

### Selection of Studies and Limiting Publication Bias

According to the recommendations by Lipsey and Wilson (2001), the following search strategy was conducted to find qualified studies. First, multiple electronic databases were searched: Ovid (including Medline, PsychINFO, and ERIC), Wiley Online Library, ScienceDirect, Academic Search Premier, EThOS, and ProQuest Dissertations & Theses. The search string comprised four elements: an attachment element, a depression element, a parent element, and an age element. For the attachment element, the following keywords were used: attachment, “parent–child relation*,” “mother–child relation*,” or “father–child relation*.” For the depression element, the following keywords were used: depressi*, dysthym*, “affective disorder*,” or “mood disorder*.” For the parent element, the keywords parent*, mother, father, caregiver, or caretaker were used. For the age element, the following keywords were used: infant, baby, babies, child*, toddler*, youth, adolescen*, “young adult,” or student. If possible, the keywords were entered in specific text fields of the databases (i.e., the title, abstract and/or keywords) to reduce the number of unqualified hits.

In systematic reviews, the aim is to include all eligible studies previously conducted (Lipsey and Wilson 2001). However, a common problem is that studies may not have been published because of non-significant or unfavorable findings, and therefore are difficult to locate, the so-called “publication or file drawer bias” (Rosenthal 1979). The consequence of publication bias is that the selection of studies is not an adequate representation of all previous studies that have been conducted. In order to prevent the problem of publication bias, we screened unpublished studies by searching the ProQuest Dissertations & Theses database and the E-theses Online Service database (EthOS). Most dissertations were publicly accessible. In case we found unpublished dissertations, we emailed the authors for the full text of the study, or ordered the study from the Proquest Dissertation Express. In addition, we emailed several attachment scholars to ask whether they knew of any unpublished articles.

The search for eligible studies was conducted by the first three authors independently. In case of any doubt, the other searchers were consulted. In total, 4892 titles were screened in the electronic databases. Further, we applied a snowball sampling method (i.e., screening the reference lists of relevant articles and the publication lists of attachment scholars) to find additional qualified studies. The initial search strategy yielded 508 studies (including reviews) of which the abstracts and methods sections were briefly read and excluded in case the study did not fit one of the inclusion criteria. Further examination of the full texts of 174 studies led to the inclusion of 124 studies, with 123 independent samples (*s*), 643 effect sizes (*k*), and a total of 54,598 participants in the current review. For a flow chart of the search procedure, see Appendix A. Appendix B presents the references of the included studies, and Appendix C presents the characteristics of the included studies.

### Coding the Studies

The first author and a research assistant coded the included studies according to the suggestions of Lipsey and Wilson (2001). The independent variable was attachment security. The dependent variable in this meta-analysis was depression. The potential moderators of the relation between attachment and depression were grouped into the following domains: study characteristics, sample characteristics, attachment, and depression characteristics.

For the study characteristics, we first coded the year of publication as a potential moderator, because we expected that the quality of recent studies was higher than the quality of older studies, as the statistical and methodological knowledge in social research has increased tremendously over the last decades. Second, the impact factor of the journal in which the study was published was coded, because the impact factor could be a first indication of study quality (Saha et al. 2003). Third, in order to assess the possible effect of publication bias on the association between attachment and depression, we coded whether the study was published in a journal or not. Fourth, at this point, it is not known whether the assumption that secure attachment relationships contribute to positive socio-emotional outcomes is true across cultures (Mesman et al. 2016), and if the strength of the association between attachment and depression varies across cultures. Therefore, the country of the research location (Anglo-Saxon and European countries vs. other countries) was coded. Finally, the study design was coded (cross-sectional vs. longitudinal design), as cross-sectional studies measure the relation between attachment and depression at one point in time (i.e., the co-occurrence of attachment insecurity and depression). Longitudinal studies take into account the developmental aspect of the association between attachment security and depression (i.e., attachment insecurity as a risk factor for depression). Also, the time (number of months) between the attachment and depression assessment was coded.

We coded various sample characteristics. First, we coded the mean age at the time of the attachment measure and the mean age of the depression measure, because in longitudinal studies, these can vary. In addition, we created three age categories: childhood sample (age range between 0 and 10 years old), pre-/early adolescence sample (age range between 9 and 15 years old), and adolescence/late adolescence (age range between 15 and 23 years old), because it is expected that the influence of parental attachment is stronger for younger children than for older children and adolescents (DeKlyen and Greenberg 2008). We choose to let the age ranges overlap, in order to increase the number of samples that could be categorized. For example, if the sample included children between 9 and 12 years old, it was categorized as a pre-/early adolescence sample. Studies with broad age ranges (e.g., 9 to 18 years old) were not categorized in this moderator.

Second, in previous meta-analyses on the relation between attachment and psychopathology, significant moderating effects of gender were found (Groh et al. 2017). Therefore, we coded whether the sample was an all-male, all-female, or mixed sample, and coded the proportions of males in the sample (continuous). Third, in line with previous meta-analyses on attachment and internalizing problems (Colonnesi et al. 2011; Madigan et al. 2013), we coded whether the families in the sample were at risk for psychopathology/attachment problems, whether it was a sample from the general population or it was a mixed sample, containing both at risk children or families and children from the general population. A sample was coded as at risk when either the parents or the children had mental health problems, when the child had experienced maltreatment or was in residential youth care, when one of the parents had died, when the children had academic risk factors (such as receiving special education), and when the sample consisted of teenage mothers. Finally, the percentage of children with Caucasian background in the sample was coded, because of possible cultural differences that may influence the association between attachment and depression (Mesman et al. 2016).

Various attachment variables were coded. First, we coded the attachment figure that was measured (parents, mothers, fathers, or general attachment representation of the child), because mothers and fathers could have unique and different influence on the development of children. Second, because the different attachment styles could influence the strength of the association between attachment and depression, we coded the type of attachment that was measured (i.e., secure, insecure-avoidant, insecure-ambivalent, insecure-disorganized, or a broad insecure measure). Third, we coded whether the attachment measure was a continuous or a categorical measure. Fourth, the type of instrument of the attachment measure (questionnaire, interview, or experiment/observation) was coded, because the different instrument may tap different elements of attachment (Bosmans and Kerns 2015). In addition, questionnaires are more sensitive to socially desirable responding than other attachment measures. Fifth, because previous meta-analyses showed moderating effects of the informant of the attachment measure on the relation between attachment and psychopathology (Colonnesi et al. 2011; Madigan et al. 2016), we coded whether the child, parent, or an observer reported on the attachment relationship. In the analyses, only the child and observer categories were included, because in only one study the parent reported on the attachment relationship.

We coded several depression variables. In line with the meta-analysis of Colonnesi et al. (2011), we coded whether the study measured depressive symptoms or clinical diagnosis of depression, and coded the informant of the depression measure (child, parent, both, or others). Because too little studies assessed depression by both parent and child or others, we only coded the child versus parent informant effect. Lastly, we coded the instrument of the depression measure (questionnaire vs. interview), because interviews are considered as more objective instruments than questionnaires (Uher and McGuffin 2010).

Ten studies that were coded by the research assistant were randomly selected and double coded by the first author. The percentages of agreement for the moderator variables ranged from sufficient for the variables impact factor (86.7%), attachment Fig. (96.7%), attachment measure (93.3%), depression instrument (96.7%), to perfect (100%) for the variables publication status, publication year, country, study design, the age variables, gender, family risk status, percentage of Caucasians in sample, type of attachment, instrument of attachment, attachment informant, depression informant, depression measure, and time between attachment and depression measure. For the calculated effect size and the sample sizes, the percentages of agreement were 96.7% and 90.0%, respectively.

### Calculations and Analyses

Effect sizes were reflected in correlation coefficients. We hypothesized that secure attachment relationships would be associated with less depression, and insecure attachment relationships to be associated with more depression. All correlations were keyed into the same direction so that these correlations could be compared to each other. A positive correlation indicated that the effect size was in line with our hypotheses, that is, attachment insecurity was hypothesized to be associated with more depression and attachment security with less depression. Cohen (1998) formulated criteria that were used for interpreting effect sizes. Effect sizes around *r* = .10 were considered as small, effect sizes around *r* = .30 as medium, and effect sizes around *r *= .50 as large.

If necessary, statistics were converted into correlational scores using the converter of Wilson (2013), and formulas from Lipsey and Wilson (2001). If a study only mentioned that an effect was not significant, the effect size was coded as zero (Lipsey and Wilson 2001). The continuous variables (publication year, impact factor, mean age of the sample, mean age at the attachment measure, mean age at the depression sample, months between attachment and depression measures, proportion of males, and proportion of Caucasians) were centered around their mean, and categorical variables were recoded into dummy variables. Extreme values of the effect sizes (> 3.29 SD from the mean; Tabachnik and Fidell 2013) were adjusted by winsorizing these outliers (i.e., replacing the outlier by the highest or lowest acceptable score falling within the normal range). Correlation coefficients *r* were recoded into Fisher *z*-values (Lipsey and Wilson 2001). In the reports on the overall relation between attachment security and depression and in the intercepts of the moderator analyses, Fisher *z*-values were transformed back into correlation coefficients for the purpose of interpretation. Standard errors and sampling variance of the effect sizes were estimated using formulas by Lipsey and Wilson (2001).

In the majority of the studies, it was possible to calculate more than one effect size, because for instance, the study reported on the correlation between different types of attachment and depression separately, or because multiple instruments and informants were used to assess attachment and depression. It is possible that effect sizes from the same study are more alike than effect sizes from different studies. Therefore, the assumption of independent effect sizes that underlie classical meta-analytic strategies was violated (Hox 2002; Lipsey and Wilson 2001). In line with recently conducted meta-analyses, we applied a multilevel approach to the current meta-analysis in order to deal with the dependency of effect sizes (Houben et al. 2015; Spruit et al. 2016). The multilevel approach accounts for the hierarchical structure of the data, in which effect sizes are nested within studies (Van Den Noortgate and Onghena 2003). Further, a multilevel meta-analysis enables using all effect sizes reported in the primary studies, so that all information is preserved and maximum statistical power is achieved (Assink et al. 2015).

We used a three-level meta-analytic model to calculate the combined effect sizes and to perform the moderator analyses, using instructions of Assink and Wibbelink (2016). Three sources of variance were modeled, including the sampling variance for the observed effect sizes (level 1), the variance between effect sizes from the same study (level 2), and the variance between the studies (level 3) (Cheung 2014). The sampling variance of observed effect sizes (level 1) was estimated by using the formula of Cheung (2014). Log-likelihood-ratio tests were performed to compare the deviance of the full model to the deviance of the models excluding one of the variance parameters, making it possible to determine whether significant variance is present at the second and third levels (Assink and Wibbelink 2016). Significant variance at level 2 or 3 indicates a heterogeneous effect size distribution, meaning that the effect sizes cannot be treated as estimates of a common effect size. In that case, we proceeded to moderator analyses, because the differences between the effect sizes may be explained by study, sample, attachment, and/or depression characteristics. Moderator analyses were only performed in case each category of the potential moderator was filled with at least three studies (Spruit et al. 2016). All significant moderators were subsequently entered in a multivariate model to examine the unique contribution in the explanation of the variance in the effect size distribution.

In case of heterogeneous effect size distribution, we are not able to test for publication bias. One of the assumptions made for statistical publication bias tests concerns the homogeneity of the data. If this assumption is not met, the publication bias tests cannot differentiate between heterogeneity and publication bias and might result in false positives or uninterpretable results (Ioannidis 2005). In the search strategy, however, we have made efforts to limit the possible effects of publication bias (see “Selection of Studies and Limiting Publication Bias”). We additionally tested in a moderator analysis of publication status whether publication bias could affect the strength of the relation between attachment and depression.

The multilevel meta-analysis was conducted in R (version 3.4.4) with the metafor package, using a multilevel random effects model (Assink and Wibbelink 2016; Spruit et al. 2016). The restricted maximum likelihood estimate was used to estimate all model parameters, and the Knapp and Hartung (2003) was used for testing individual regression coefficients of the meta-analytic models and for calculating the corresponding confidence intervals (see also Assink et al. 2015; Houben et al. 2015; Assink and Wibbelink 2016).

## Results

The meta-analysis included 124 studies, with 123 independent samples (*s*), 643 effect sizes (*k*), and a total of 54,598 participants. The results of the multilevel meta-analysis on the relation between attachment security and depression in children and adolescents are presented in Table 1. The overall association between attachment and depression can be found in this table, as well as the results of the moderator analyses.

**Table 1:** The overall results and moderator effects of the relation between attachment and depression

	*s*	*k*	β_0_	Mean *r*	*t*_0_			
Overall association attachment and depression	123	643	0.324	.313	22.177			
Moderator variables	*s*	*k*	β_0_	Mean *r*	*t*_0_	β_1_	t_1_	*F*(df1, df2)
Study characteristics								
Year of publication (cont.)	123	643	0.324	.313	22.096***	− 0.001	− 0.661	*F*(1,641) = 0.437
Impact factor (cont.)	105	541	0.327	.316	21.178***	− 0.024	− 2.331*	*F*(1,539) = 5.435*
Publication status	123	643						*F* (1,641) = 0.879
Published (RC)	106	541	0.325	.314	20.425***			
Not published	17	102	0.319	.309	8.405***	− 0.006	− 0.152	
Country	123	613						*F*(1,611) = 12.028***
Western (RC)	112	554	0.316	.306	21.419***			
Non-Western	13	59	0.393	.374	15.932***	0.077	3.468***	
Study design	123	643						*F*(1,641) = 39.184***
Cross-sectional (RC)	97	462	0.361	.346	23.661***			
Longitudinal	32	181	0.203	.200	8.502***	− 0.158	− 6.260***	
Time between attachment and depression measure (cont.)	123	637	0.330	.319	24.127***	− 0.002	− 5.417***	*F*(1,635) = 29.344***
Sample characteristic								
Mean age attachment measure (cont.)	112	609	0.328	.317	22.069***	0.010	2.835**	*F*(1,607) = 8.036**
Mean age depression measure (cont.)	112	609	0.327	.316	21.276***	− 0.003	− 0.741	*F*(1,607) = 0.549
Age category	68	308						*F*(2,305) = 3.802*
Child sample (RC)	8	31	0.181	.179	3.335***			
Pre-/early adolescence	34	181	0.341	.328	15.293***	0.160	2.728**	
(Late) adolescence	29	96	0.312	.302	12.437***	0.131	2.186*	
Gender	120	627						*F*(2,624) = 8.036**
All male (RC)	8	33	0.341	.328	9.914***			
All female	21	79	0.403	.383	14.040***	0.063	2.086^*^	
Mixed	101	627	0.314	.304	20.948	− 0.027	− 0.777	
Family risk status	123	643						*F*(2,640) = 0.467
No risk (RC)	72	511	0.330	.319	19.656***			
Risk	19	87	0.318	.308	8.151***	− 0.013	− 0.301	
Mixed	12	45	0.283	.276	6.020***	− 0.047	− 0.950	
Proportion Caucasian (cont.)	68	322	0.301	.292	15.420***	− 0.058	− 1.034	*F*(1,320) = 1.070
Attachment variables								
Attachment figure	123	643						*F*(3,639) = 0.278
Parents (RC)	53	207	0.314	.304	16.302***			
Mother	62	234	0.332	.320	17.784***	0.018	0.822	
Father	31	132	0.326	.315	15.881***	0.012	0.519	
General attachment	14	70	0.330	.319	9.207***	0.016	0.419	
Attachment style	82	521						*F*(4,516) = 5.067***
Secure (RC)	42	264	0.307	.298	13.575***			
Avoidant	29	57	0.273	.266	9.680***	− 0.033	− 1.173	
Ambivalent	32	55	0.320	.310	11.245***	0.013	0.442	
Disorganized	12	27	0.379	.362	8.959***	0.073	1.718^+^	
Insecure	35	118	0.353	.339	15.013***	0.046	3.537***	
Attachment measure	123	643						*F*(1,641) = 13.358***
Continuous (RC)	112	552	0.341	.328	23.758***			
Categorical	24	91	0.229	.225	7.894***	− 0.112	3.655***	
Instrument attachment	123	643						*F*(2,640) = 20.381***
Questionnaire (RC)	100	518	0.358	.343	25.652***			
Interview	11	78	0.156	.155	3.787***	− 0.203	− 4.748***	
Observation/experiment	14	47	0.162	.161	4.030***	− 0.197	− 4.657***	
Informant attachment	123	642						*F*(1,640) = 12.687***
Child (RC)	112	605	0.338	.326	23.484***			
Observator	12	37	0.165	.164	3.509***	− 0.173	− 3.562***	
Depression variables								
Depression measure	123	643						*F*(1,641) = 0.039
Symptoms (RC)	116	608	0.325	.314	21.618***			
Clinical diagnosis	11	35	0.315	.305	6.900***	− 0.009	− 0.196	
Instrument depression	122	639						*F*(1,637) = 0.479
Questionnaire (RC)	117	600	0.326	.315	21.516***			
Interview	14	39	0.296	.288	6.977	− 0.030	− 0.692	
Informant depression	121	627						*F*(1,625) = 2.442
Child (RC)	120	597	0.327	.316	22.375***			
Parent	5	30	0.273	.266	7.458***	− 0.054	− 1.563	

*s* number of independent studies, *k* number of effect sizes, *t*0 difference in mean r with zero, *t*1 difference in mean *r* with reference category, *mean r* mean effect size (*r*), *F(df1, df2)* omnibus test, *cont*. continuous variable, *RC* reference category

*p < .05; **p < .01; ***p < .001; ^+^p < .10

### Overall Relation Between Attachment Security and Depression

Overall, a significant, moderate association was found between attachment and depression in children and adolescents, *r* = .31, *p* < .001; 95% CI [0.29, 0.34], indicating that, in line with the expectations, attachment security is negatively correlated with depression, and attachment insecurity is positively correlated with depression. More specifically, the association between attachment and depression was significant for both cross-sectional (*r* = .35, *p* < .001) and longitudinal studies (*r* = .20, *p* < .001), indicating that attachment insecurity both co-occurs with and precedes depression.

The likelihood ratio test comparing models with and without between-study variance (level 3) showed that significant variance was present at the between-study level, σ^2^ level 3 = 0.02, *χ*^*2*^(1) = 324.86, *p* < .0001. The variance between the effect sizes within studies (level 2) was significant as well, σ^2^ level 2 = 0.01, *χ*^*2*^(1) = 1995.78, *p* < .0001, indicating a heterogeneous effect size distribution. Of the total effect size variance, 6.3% was accounted for the sampling variance (level 1), 27.3% for the variance between effect sizes within studies (level 2), and 66.4% for the variance between studies (level 3). Because of this heterogeneous effect size distribution, we conducted moderator analyses to examine whether the strength of the association between attachment security and depression is affected by study, sample, attachment, and depression characteristics. We did not test for publication bias, because these tests require homogeneous data.

### Moderators of the Relation Between Attachment Security and Depression

#### Study Characteristics

The impact factor of the journal significantly moderated the strength of the relation between attachment and depression. Studies that were published in journals with higher impact factors yielded smaller effect sizes. In addition, the county of the study was a significant moderator of the association between attachment and depression. Larger effect sizes were found in studies from non-Western countries, compared to studies from Anglo-Saxon and European countries. Moreover, the study design significantly influenced the strength of the relation. Smaller effect sizes were found in longitudinal studies compared to cross-sectional studies. Because study design was a significant moderator, we tested whether the amount of time between the attachment and depression assessment influenced the strength of the relation. As expected, we found that the more time between the attachment and depression measure, the smaller the effect size. The year of publication and the publication status (published in a journal or not) did not moderate the association.

#### Sample Characteristics

Various sample characteristics moderated the association between attachment and depression. First, the child’s age during the attachment measurement influenced the strength of the association between attachment and depression: in older samples there were stronger associations. To further estimate this moderator effect of age, we tested the moderating effect of the categorical age variable (i.e., childhood vs. pre-/early adolescence vs. adolescence/late adolescence). The effect sizes in childhood samples (< 10 years of age) were significantly smaller than in the adolescence samples. Second, the gender of the samples significantly moderated the relation between attachment and depression. All-female samples yielded significantly larger effect sizes than all-male samples. Finally, the mean age at the depression assessment and the family risk status were no significant moderators.

#### Attachment Variables

All types of attachment styles (i.e., secure, insecure-avoidant, insecure-ambivalent, insecure-disorganized, and a broad insecure category) were significantly associated to depression. The insecure styles were positively associated with depression, the secure style was negatively associated with depression. However, the type of attachment style influenced the strength of the relation. The broad insecure measures yielded significantly larger effect sizes than the secure measures. The effect sizes in the other insecure attachment measures did not differ significantly from the secure attachment measure, although insecure-disorganized attachment almost reached significance (*p* > .10) for larger effect sizes, compared to the secure measure. Further, smaller effect sizes were found for categorical attachment measures compared to continuous attachment measures. In addition, the type of instrument of the attachment assessment was a significant moderator. Larger effect sizes were found in questionnaire assessments compared to interviews and observations/experiments. Moreover, the informant of the attachment measure moderated the relation between attachment and depression. Larger effect sizes were found in case the child was the informant compared to when an observer was the informant. Finally, the attachment figure (i.e., parents, mothers, fathers, or general attachment representations) did not influence the strength of the correlation between attachment and depression.

#### Depression Variables

None of the depression variables were significant moderators. The depression measure (symptoms or clinical diagnoses), the depression instrument (questionnaire or interview), and the depression informant (child or parent) did not influence the relation between attachment security and depression.

### Multivariate Model

We examined the unique contribution of the significant moderators to the variance in effect sizes by applying a multivariate model. We included all moderators that were significant in the bivariate models, except for the variables time between the attachment and depression and the categorical age variable, because of overlap with the other constructs. The results of the multivariate model can be found in Table 2.

**Table 2:** Results of the multivariate model

	*s*	*k*	β	*t*	*F*(df1, df2)	σ^2^ level 3	σ^2^ level 2
Multivariate model	66	404			*F*(9,394) = 9.390***	0.018	0.006
Intercept			0.242	2.984**			
Impact factor			0.009	0.725			
Non-Western countries			0.077	3.910***			
Longitudinal design			− 0.137	− 4.396***			
Age at attachment measure			− 0.008	− 1.315			
Female sample			0.113	3.668***			
Insecure attachment			0.047	3.332***			
Categorical measure attachment			0.005	0.126			
Questionnaire attachment			0.197	3.902***			
Child informant attachment			0.006	0.082			

*s* number of independent studies, *k* number of effect sizes, *F(df1, df2)* omnibus test, *σ*^*2*^*level 3* variance at the between-study level, *σ*^*2*^*level 2* variance at the between effect size level

**p < .01; ***p < .001

We found that the country of the study, the design of the study, female gender, insecure attachment, and the type of instrument to assess attachment uniquely contributed to the explanation of variance, controlling for the effect of the other variables in the model. Longitudinal studies yielded smaller effect sizes. Larger effect sizes were found in non-Western countries, female samples, broad insecure attachment measures, and attachment questionnaires. The impact factor of the journal in which the study was published, the age at the attachment measurement, the type of attachment measure (categorical or continuous), and the informant of the attachment measure did no longer contribute to explained variance in the multilevel model.

## Discussion

### Overall Association Between Attachment and Depression

The current multilevel meta-analysis aimed to test the association between attachment security and depression in youth using a multilevel approach, and to examine potential moderators of this association. Overall, we found a significant, moderate correlation of *r *= .31 between attachment and depression, indicating that secure attachment is negatively and insecure attachment is positively associated to depression in youth. This result is in line with the previous meta-analysis on the association between attachment and anxiety, yielding an overall effect size of *r *= .30 (Colonnesi et al. 2011), and the latest meta-analysis on the association between attachment and internalizing problems of *r *= .28 (Madigan et al. 2016). Moreover, the current meta-analytic study showed a significant association between attachment and depression symptoms in both cross-sectional and longitudinal studies. Kraemer et al. (2001) have provided a framework for understanding the meaning of associations. Using the terminology of Kraemer et al. (2001), it can be concluded from this meta-analysis that attachment and depression are correlates (i.e., that there is co-occurrence between these two variables), and that attachment insecurity is shown to precede depressive symptoms (i.e., attachment insecurity is a risk factor for depression).

Bowlby’s attachment theory (1969) provided a theoretical framework to understand the relation between attachment and depression. He stated that the attachment experiences with caregivers shape the internal working model of the child, that is, generalized expectancies about the self and others (Bowlby 1969). These negative cognitions of the self and others may in turn result in depressive symptoms (Dozois and Beck 2008). Moreover, insecure attachment is found to be related to elevated stress levels and to lower emotion regulation in children (Brenning et al. 2012; Brumariu 2015; Zimmer-Gembeck et al. 2017), which may trigger depressive symptoms (Banasr et al. 2017; Juster et al. 2010; Malik et al. 2015). Finally, the association between attachment and depression could be explained by the intergenerational transmission of shared genetic and/or social-ecological vulnerability for both attachment problems and depression (Brenning et al. 2011).

### Moderators of the Attachment–Depression Association

In the current meta-analysis, we found significant variance both at the between and at the within-study level, indicating that the effect size distribution was heterogeneous, and that moderating variables may explain differences in the strength of the overall effect size. Moderator analyses showed significant effects of impact factor of the journal in which the study was published, country where the study had been carried out (Anglo-Saxon and European countries vs. other countries), design of the study (cross-sectional vs. longitudinal), time between the attachment and depression measurement, age and gender of the sample, attachment style (i.e., secure and different types of insecure styles), attachment measure (continuous vs. categorical), the instrument to assess attachment (questionnaire vs. interview vs. observation/experiment), and the informant of the attachment (child vs. observer). In the multivariate analysis including all significant moderators, country of the study, design of the study, gender, attachment style, and the type of instrument to assess attachment uniquely contributed to the explanation of variance.

Significantly smaller effect sizes were found in studies conducted in Anglo-Saxon and European countries compared to studies in other (non-Western) countries. This moderating effect could be explained by the fact that the majority of the non-Western studies were conducted in developing countries or were considered to be developing countries during the time of the study. Mesman et al. (2016) have suggested that, because of socioeconomic difficulties, parents in developmental countries are less sensitive to their children, and therefore, the incidence of insecure attachment relationships may be somewhat larger in developing countries. As a result, the variance of insecure attachment could be larger in non-Western samples, causing larger effect sizes between attachment and depression. This explanation has little empirical support, because of the lack of studies in developmental countries. However, in Western samples, the incidence of insecure attachment relationships was found to be higher in low SES samples compared to average SES samples (Van IJzendoorn et al. 1999). Other explanations for differences in effect sizes between Western and non-Western countries include variability associated with measurement, such as the overall lack of validation of instruments in non-Western samples, with potentially biased outcomes as a result.

Studies with cross-sectional designs yielded significantly larger effect sizes compared to longitudinal studies. In general, cross-sectional correlations tend to be higher than longitudinal correlations, possibly because of less interference of factors that may impact on both attachment and depression and the increased likelihood of bidirectional effects in social phenomena during a restricted period of time. From a developmental perspective, it is important to realize that attachment relationships and representations are not static characteristics. Although relatively stable, new attachment experiences or changed conditions (such as life events, or improved parental functioning) can alter the quality of the attachment relationship and the internal working model of the child (Fraley 2002; Waters et al. 2000), explaining the smaller effect sizes in longitudinal studies. Further, the moderating effect of study design partly explains that previous meta-analyses found small correlations between attachment in infancy and childhood internalizing problems (*r *= .07, Groh et al. (2012) and *r *= .18, Madigan et al. 2013). Although the infant years are of great importance in the foundation of a children’s attachment representation, attachment does not stop after infancy (Bowlby 1969), and is likely to change, for instance, because of new attachment experiences with those who might become attachment figures (Fraley 2002).

In samples with only females, larger effect sizes were found compared to samples with only males, implicating that there is a stronger association between attachment security and depression in girls then in boys. Although in line with the expectations of DeKlyen and Greenberg (2008) that attachment insecurity has a stronger effect on internalizing problems in girls than boys, this moderator effect has not yet been established in other meta-analyses on attachment and internalizing problems (Groh et al. 2012; Madigan et al. 2013, 2016). The absence of gender moderators in other quantitative reviews could be explained by the multilevel nature of the current meta-analysis, allowing for more comprehensive moderator analyses, resulting in more statistical power to detect moderators of the overall mean effect size (Assink et al. 2015). The number of all-male and all-female samples in other meta-analyses may have been too small to make meaningful comparisons, as suggested by Groh et al. (2012).

From a theoretical perspective, the moderating effect of gender might be explained by differences in the impact of social and relational factors on psychological functioning between boys and girls. Attachment experiences shape the quality of future social relationships of youth (Bowlby 1969). Girls desire more closeness and dependency on social relationships than boys, and also worry more about abandonment, loneliness, and social pain (Rose and Rudolph 2006). The quality of social support has shown to be especially influential in the wellbeing of girls (Rueger et al. 2010). Therefore, it is not unlikely that insecure attachment has a stronger effect on depressive symptoms in girls compared to boys.

The style of attachment was a significant moderator of the correlation between attachment and depression. Studies using a broad insecure attachment measure (e.g., the Alienation subscale of the Inventory of Parent and Peer Attachment, Armsden and Greenberg 1987) yielded stronger effect sizes compared to studies using a secure attachment measure (for example the Security Scale; Kerns et al. 1996). A secure attachment relationship serves as a protective factor for psychopathology, whereas insecure attachment relationships are considered to be a risk factor (Colonnesi et al. 2011; Groh et al. 2012; Madigan et al. 2013). Risk factors generally outweigh buffering effects of protective factors (Baumeister et al. 2001), which could explain why we found larger effect sizes for insecure attachment measures, compared to secure attachment measures. In addition, we found a moderating trend (*p *< .10) suggesting that disorganized attachment measures produced larger effect sizes than secure attachment measures. Disorganized attachment is perceived as the most insecure attachment type and most frequently linked to psychopathology (Groh et al. 2017; Lyons-Ruth and Jacobvitz 2008; Madigan et al. 2016), which might explain why larger (although only trend-significant) effect sizes were found for disorganized attachment.

Finally, we found significant larger effect sizes for the association between attachment and depression when attachment was measured by a self-report questionnaire compared to interviews or observations and experiments. The most important explanation for this moderator is that in most of the studies, depression was also assessed with a self-report questionnaire. Constructs that are measured by the same type of instrument (i.e., self-report questionnaires) generally show larger accordance than if constructs are measured by multiple types of instruments, also referred to as common method variance (Podsakoff et al. 2003).

### Limitations

There are several limitations that need to be mentioned. First, we tested whether there is an association between attachment security and depression, but this does not necessarily mean causality (Kreamer et al. 2001). The most dominant theories on the relation between attachment and depression assume causality (i.e., attachment insecurities be in the root of depressive symptoms). Other theories do not assume causality, for example, explaining the association between attachment and depression by a shared risk environment, accounting for both the presence of depression and attachment insecurity in children. The results from the longitudinal studies in the current meta-analysis imply that attachment insecurity is a risk factor for depression. However, we have not focused on whether attachment insecurity is a *causal* risk factor (i.e., a risk factor that, when changed, is shown to change the outcome; Kreamer et al. 2001) for depression. Although assessing the effects of attachment-based intervention on depression by means of (quasi-)experimental studies or multiple case studies is not an ultimate test of causality, it could provide some indication that attachment problems contribute to the onset of depression. Therefore, it is recommended that future studies on the effect of attachment-based interventions test whether changes in attachment security are related to changes in depressive symptoms.

Second, we cannot rule out a potential publication bias in our results. Due to the heterogeneous effect size distribution of the data, statistical methods to detect the presence of publication bias could not be applied, because they become biased in case of heterogeneity (Ioannidis 2005). If publication bias would be present in the current results, the relation between attachment and depression might be overestimated. However, in the majority of the studies testing the association between attachment and depression was only one of the research aims, which makes it unlikely that the whole study would not be published in case of an insignificant result.

### Implications for Research and Clinical Practice

The current meta-analytic review offers several implications for clinical practice. First and foremost, we found a significant association between attachment and depression in cross-sectional studies, which suggest that youth with attachment problems often experience depressive symptoms and vice versa. As attachment insecurity often occurs in depressive youth, it is important to screen for attachment insecurity in youth who are referred to psychological treatment because of depressive symptoms. Second, in youth who experience both attachment insecurity and depression, attachment insecurity may be targeted by the therapeutic interventions as well. The most common and best researched treatment for depression is cognitive behavioral therapy (CBT), which often shows moderate effects on depression (Hofmann et al. 2012; Zhou et al. 2015). CBT aims to reduce depressive symptoms, for example, by learning to recognize and solve problems, performing behavioral experiments, and challenging irrational cognitions. Bosmans (2016) argued that the limited treatment effects of CBT could be explained by the lack of focus on attachment problems in CBT in its current form. In CBT, parents are often involved in the treatment (for example, by practicing the behavioral experiments together with the child). However, it may be necessary to improve the attachment relationship first, in order for the parent to be able to provide the trusted secure base and secure haven the child needs for the CBT exercises. Bosmans (2016) advocated attachment-based family therapy (ABFT; Diamond et al. 2003) and attachment-focused cognitive bias modification (CBM_A; De Winter et al. 2017) as promising interventions for depression in youth, and suggested to incorporate attachment-focused techniques in CBT practice, because attachment insecurity often occurs in depressive youth (Bosmans 2016).

ABFT, CBM_A, and attachment-focused CBT are relatively new interventions, and future research should investigate whether these interventions are superior to CBT. Notably, there is empirical evidence showing that a focus on relational aspects of depressive symptoms might be fruitful. For example, interpersonal Psychotherapy (IPT; Weissman et al. 2008) is a psychological intervention specifically developed for the treatment of major depression, and is based on interpersonal theory, including Bowlby’s (1969) attachment theory. IPT focusses on current interpersonal relationships, and intervenes in the social dysfunction associated with depression. A recent meta-analysis showed that IPT is more effective in reducing depression in youth than CBT (Zhou et al. 2015). All in all, there is growing evidence that interventions for depression should be informed by attachment theory.

The current study also offers implications for future studies. We found a significant correlation between attachment and depression in longitudinal studies, and thus, that attachment insecurity is a risk factor for depression. However, we did not test whether attachment insecurity is also a *causal* risk factor (Kraemer et al. 2001) for depression, meaning that we did not examine whether depression can be prevented if attachment insecurity in the early years is treated. Although several attachment-based interventions have shown to be effective in reducing internalizing problems (e.g., Allen et al. 2014; Stams et al. 2001), most studies focus on the prevention of externalizing problems with the use of attachment-based interventions. Therefore, future studies should examine whether treating attachment insecurity can prevent depression later on in life.

Second, some hypotheses on the relation between attachment security and depression could not be tested. For example, an in-depth exploration of cultural differences that may moderate the association between attachment and depression was not conducted, due to the limited number of non-Western studies. In line with Mesman et al. (2016), we emphasize that future studies on the relation between attachment security and depression in children should be conducted in other cultures than the Angel-Saxon and European culture. Further, the current study gave some indication that disorganized attachment has the strongest association with depression. However, this needs to be further explored. Finally, it is recommended to assess potential interactions between moderators in future meta-analyses. For example, some studies showed that the unique influence of mothers and fathers on depression could be different for boys and girls (Liu 2006; De Minzi 2010). Assessing interactions between moderators on a meta-analytic level could lead to further understanding of the association between attachment and depression in youth.

## Conclusion

The current meta-analytic study on the association between attachment and depression in youth yielded a moderate effect size of *r *= .31. Attachment insecurity and depressive symptoms in youth are associated in both longitudinal and cross-sectional studies, and therefore we argue that attachment problems and depressive symptoms co-occur and that attachment insecurity is a risk factor for depression. The association between attachment security and depression in youth can be explained at different levels, including genetic factors (through shared genetic vulnerability for attachment problems and depression), cognitive factors (in which the internal working model of the child is the base of dysfunctional cognitions), socio-emotional factors (including emotion regulation and social skills), and factors at the level of culture and society (for example, though a shared socio-ecological risk environment). In addition, the current meta-analytic study provided insights into the moderators of the association between attachment and depression. Larger effect sizes were found in cross-sectional and non-Western studies, in female samples, in questionnaires, and in broad insecure attachment measures. Clinical practice should incorporate attachment theory to increase the effectiveness of depression interventions. It is recommended to further explore the relation between attachment security and depression in future studies, for example, by conducting studies in non-Western countries, applying longitudinal mediation models, and by testing interactions between potential moderators.

## Electronic supplementary material

Below is the link to the electronic supplementary material.

Supplementary material 1 (DOCX 175 kb)
