# Supplementary material for: The Relation Between Attachment and Depression in Children and Adolescents: A Multilevel Meta-Analysis
Source: Clin Child Fam Psychol Rev. 2019 Aug 7;23(1):54–69. doi: 10.1007/s10567-019-00299-9 (PMC7000490; doi:10.1007/s10567-019-00299-9)
Supplement: Supplementary file 1 — Supplementary material 1 (DOCX 175 kb) [file 10567_2019_299_MOESM1_ESM.docx]

**Supplemental material**

Article title: The Relation between Attachment Security and Depression in Children and Adolescents: a Multilevel Meta-Analysis

Journal name: Clinical Child and Family Psychology Review

Author names: Spruit, Goos, Weenink, Rodenburg, Niemeyer, Stams, & Colonnesi

Corresponding author: Dr. Cristina Colonnesi. Research Institute of Child Development and Education, Faculty of Social and Behavioural Sciences, University of Amsterdam. [C.colonnesi@uva.nl](mailto:C.colonnesi@uva.nl)

**Appendix A Flowchart of the search**
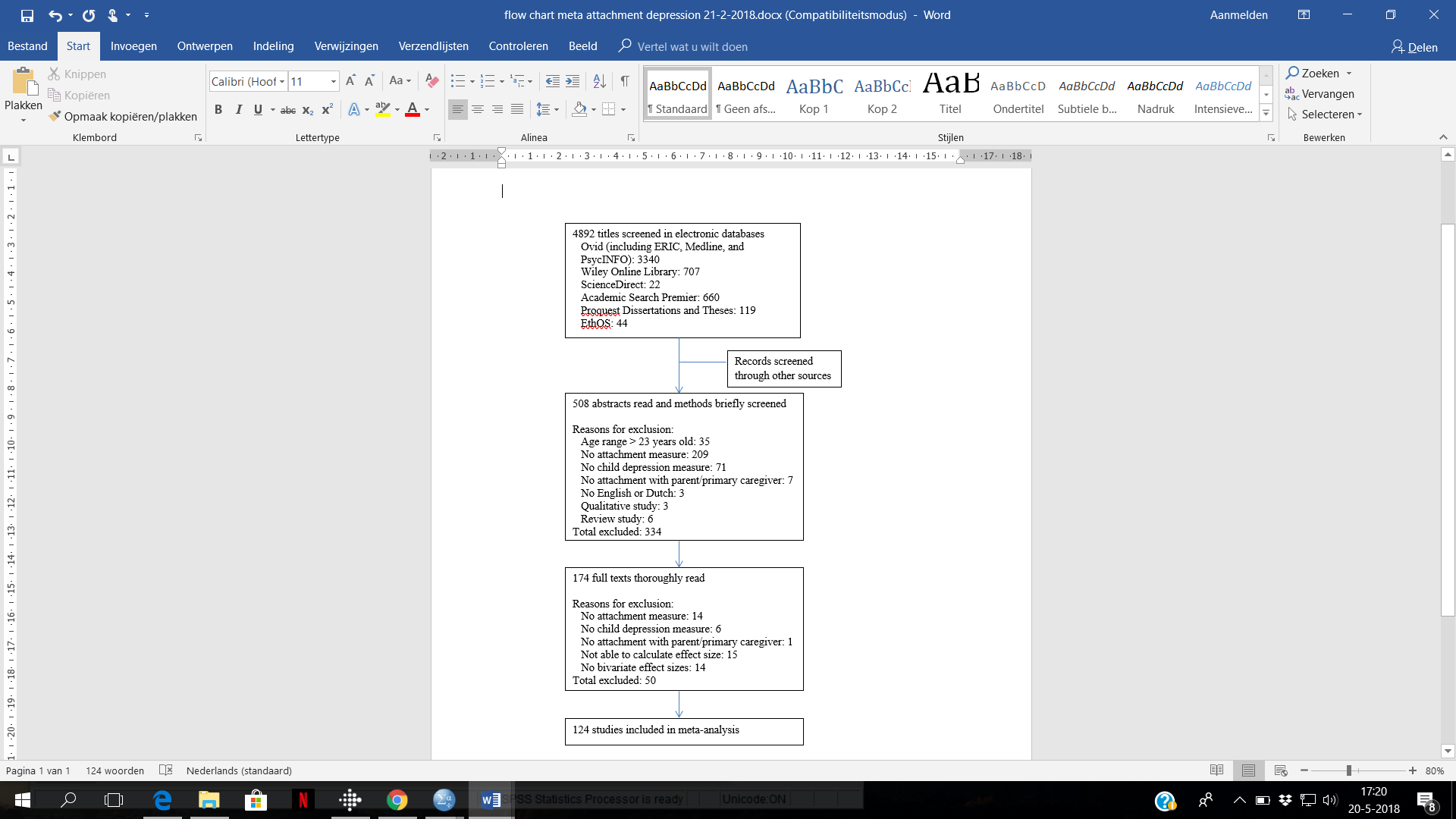


**Appendix B The Studies Included in the Meta-Analysis**

1. Abela, J. R. Z., Hankin, B. L., Haigh, E. A. P., Adams, P., Vinokuroff, T., & Trayhern, L. (2005). Interpersonal vulnerability to depression in high risk children: The role of insecure attachment and reassurance seeking. *Journal of Clinical Child and Adolescent Psychology, 34,* 182-192. https://doi.org/10.1207/s15374424jccp3401_17
2. Agerup, T., Lydersen, S., Wallander, J., & Sund, A. M. (2015). Associations between parental attachment and course of depression between adolescence and young adulthood. *Child Psychiatry & Human Development*, *46*, 632-642. https://doi.org/10.1007/s10578-014-0506-y
3. Aifuwa, S. (2016). *Effects of child-parent attachment and God attachment on depression in adolescent Christians* (Doctoral dissertation). Retrieved from http://digitalcommons.liberty.edu/doctoral/1355/.
4. Allen, J. P., Porter, M., McFarland, C., McElhaney, K. B., & Marsh, P. (2007). The relation of attachment security to adolescents’ paternal and peer relationships, depression, and externalizing behavior. *Child Development, 78,* 1222-1239. https://doi.org/10.1111/j.1467-8624.2007.01062.x
5. Armsden, G. C., & Greenberg, M. T. (1987). The inventory of parent and peer attachment: Individual differences and their relationship to psychological well-being in  adolescence. *Journal of Youth & Adolescence, 16,* 427 - 454. https://doi.org/10.1007/bf02202939
6. Armsden, G. C., McCauley, E., Greenberg, M. T., Burke, P. M., & Mitchell, J. R. (1990). Parent and peer attachment in early adolescent depression. *Journal of Abnormal Child Psychology, 18,* 683-697. https://doi.org/10.1007/bf01342754
7. Bámaca-Colbert, M. Y., Umaña-Taylor, A. J., & Gayles, J. G. (2012). A developmental-contextual model of depressive symptoms in Mexican-origin female adolescents. *Developmental Psychology*, *48*, 406. https://doi.org/10.1037/a0025666
8. Bauman, L. J., Foster, G., Johnson Silver, E., Berman, R., Gamble, I., & Muchaneta, L. (2006). Children caring for their ill parents with HIV/AIDS. *Vulnerable Children and Youth Studies*, *1*, 56-70. https://doi.org/10.1080/17450120600659077
9. Bennett (2004). *The role of attachment in the relationship between maternal and childhood depressive symptomatology* (Doctoral dissertation). Retrieved from https://repositories.lib.utexas.edu/bitstream/handle/2152/1177/bennettl81291.pdf.
10. Bogard, K. L. (2005). Affluent adolescents, depression, and drug use: The role of adults in their lives. *Family Therapy*, *32*, 95.
11. Borelli, J. L., Crowley, M. J., David, D. H., Sbarra, D. A., Anderson, G. M., & Mayes, L. C. (2010). Attachment and emotion in school-aged children. *Emotion*, *10*, 475. https://doi.org/10.1037/a0018490
12. Borelli, J. L., Smiley, P. A., Rasmussen, H. F., Gómez, A., Seaman, L. C., & Nurmi, E. L. (2017). Interactive effects of attachment and FKBP5 genotype on school-aged children’s emotion regulation and depressive symptoms. *Behavioural brain research*, *325*, 278-289. https://doi.org/10.1016/j.bbr.2016.07.035
13. Bosmans, G., Poiana, N., Van Leeuwen, K., Dujardin, A., De Winter, S., Finet, C., Heylen, J. & Van de Walle, M. (2016). Attachment and depressive symptoms in middle childhood: The moderating role of skin conductance level variability. *Journal of Social and Personal Relationships*, *33*, 1135-1148. https://doi.org/10.1177/0265407515618278
14. Bosquet, M., & Egeland, B. (2006). The development and maintenance of anxiety symptoms from infancy through adolescence in a longitudinal sample. *Development and psychopathology*, *18*, 517-550. https://doi.org/10.1017/s0954579406060275
15. Branje, S. J. T., Hale, W. W. III., Frijns, T. Meeus, W. H. J. (2010). Longitudinal associations between perceived parent-child relationship quality and depressive symptoms in adolescence. *Journal of Abnormal Child Psychology, 38,* 751-763. https://doi.org/10.1007/s10802-010-9401-6
16. Brenning, K., Soenens, B., Braet, C., & Bosmans, G. (2011). The role of depressogenic personality and attachment in the intergenerational similarity of depressive symptoms: A study with early adolescent and their mothers. *Personality and Social Psychology Bulletin, 37*, 284. https://doi.org/10.1177/0146167210393533
17. Brenning, K. M., Soenens, B., Braet, C., & Bosmans, G. (2012). Attachment and depressive symptoms in middle childhood and early adolescence: Testing the validity of the emotion regulation model of attachment. *Personal Relationships, 19*, 445-464. https://doi.org/10.1111/j.1475-6811.2011.01372.x
18. Brenning, K., Soenens, B., Braet, C., & Bal, S. (2012). The role of parenting and mother-adolescent attachment in the intergenerational similarity of internalizing symptoms. *Journal of Youth and Adolescence, 41,* 802-816. https://doi.org/10.1007/s10964-011-9740-9
19. Brenning, K., Soenens, B., Braet, C., & Beyers, W. (2013). Longitudinal dynamics of depressogenic personality and attachment dimensions in adolescence: An examination of associations with changes in depressive symptoms. *Journal of Youth and Adolescence, 42,* 1128-1144. https://doi.org/10.1007/s10964-012-9879-z
20. Brodie, C. S. (2004). *Adolescent-parent attachment, emotion regulation and interpersonal competence in adolescence: a study of a psychiatric and a non-clinical population* (Doctoral dissertation). Retrieved from https://www.era.lib.ed.ac.uk/handle/1842/23832.
21. Buist, K. L., Dekovič, M., Meeus, W., & Van Aken, M. A. G. (2004). Gehechtheid en internaliserend en externaliserend probleemgedrag bij adolescenten. *Kind en Adolescent,* *25*, 132-149. https://doi.org/10.1007/bf03060909
22. Bureau, J. F., Easterbrooks, M. A. , & Lyons-Ruth, K. (2009). Maternal depressive symptoms in infancy: Unique contribution to children’s depressive symptoms in childhood and adolescent? *Development and Psychopathology, 21*, 519-537. https://doi.org/10.1017/s0954579409000285
23. Burge, D., Hammen, C., Davila, J., & Daley, S. E. (1997). The relationship between attachment cognitions and psychological adjustment in late adolescent women. *Developmental Psychology, 9*, 151-167. https://doi.org/10.1017/s0954579497001119
24. Cawthorpe, D., West, M., & Wilkes, T. (2004). Attachment and depression: The relationship between the felt security of attachment and clinical depression among hospitalized female adolescents. *The Canadian Child and Adolescent Psychiatry Review, 13*, 31-35.
25. Chaowiang, K. O. (2008). *A Path Analytic Model of Depressive Symptoms Among Thai Adolescents* (Doctoral dissertation). Retrieved from ProQuest Dissertations and Theses.
26. Chédebois, L., Régner, I., Van Leeuwen, N., Chauchard, E., Séjourné, N., Rodgers, R., & Chabrol, H. (2009). Relative contributions of acculturation and psychopathological factors to cannabis use among adolescents from migrant parents. *Addictive Behaviors*, *34*, 1023-1028. https://doi.org/10.1016/j.addbeh.2009.06.011
27. Chesmore, A. A., Weiler, L. M., Trump, L. J., Landers, A. L., & Taussig, H. N. (2017). Maltreated children in out-of-home care: the relation between attachment quality and internalizing symptoms. *Journal of child and family studies*, *26*, 381-392. https://doi.org/10.1007/s10826-016-0567-6
28. Cole-Detke, H., & Kobak, R. (1996). Attachment processes in eating disorder and depression. *Journal of Consulting and Clinical Psychology, 64,* 282-290.
29. Constantine, M. G. (2006). Perceived family conflict, parental attachment, and depression in African American female adolescents. *Cultural Diversity and Ethnic Minority Psychology, 12,* 697-709. https://doi.org/10.1037//0022-006x.64.2.282
30. Cooper-Newark, S. E. (2015). *Are attachment avoidance and anxiety associated with depression through specific interpersonal problem types?: a cross sectional study in older adolescents* (Doctoral dissertation, University of Surrey). Retrieved from http://epubs.surrey.ac.uk/808530/.
31. Cotterell, J. L. (1992). The relation of attachments and supports to adolescent well-being and school adjustment. *Journal of Adolescent Research*, *7*, 28-42. https://doi.org/10.1177/074355489271003
32. Crittenden, P., Robson, K., & Tooby, A. (2015). Validation of the School-age Assessment of Attachment in a short-term longitudinal study. *Clinical child psychology and psychiatry*, *20*, 348-365. https://doi.org/10.1177/1359104515589641
33. Demidenko, N., Manion, I., & Lee, C. M. (2015). Father–daughter attachment and communication in depressed and nondepressed adolescent girls. *Journal of Child and Family Studies*, *24*, 1727-1734. https://doi.org/10.1007/s10826-014-9976-6
34. De Minzi, M. C. R., & Richaud, C. (2006). Loneliness and depression in middle and late childhood: The relationship to attachment and parental styles. *Journal of Genetic Psychology, 167,* 189-210. https://doi.org/10.3200/gntp.167.2.189-210
35. Dhillon, R., & Kanwar, P. (2015). Relationship of perceived parental attachment with internalizing problems among adolescents. *Indian Journal of Health and Wellbeing*, *6*, 171.
36. Diamond, G. S., Reis, B. F., Diamond, G. M., Siqueland, L., & Isaacs, L. (2002). Attachment-based family therapy for depressed adolescents: A treatment development study. *Journal of the American Academy of Child & Adolescent Psychiatry*, *41*, 1190-1196.
37. Dujardin, A., Santens, T., Braet, C., De Raedt, R., Vos, P., Maes, B., & Bosmans, G. (2016). Middle childhood support‐seeking behavior during stress: Links with self‐reported attachment and future depressive symptoms. *Child Development*, *87*, 326-340. https://doi.org/10.1111/cdev.12491
38. Duchesne, S., & Ratelle, C. F. (2014). Attachment security to mothers and fathers and the developmental trajectories of depressive symptoms in adolescence: Which parent for which trajectory? *Journal of youth and adolescence*, *43*, 641-654. https://doi.org/10.1007/s10964-013-0029-z
39. Eberhart, N. K., & Hammen, C. L. (2006). Interpersonal predictors of onset of depression during the transition to adulthood. *Personal Relationships*, *13*, 195-206. https://doi.org/10.1111/j.1475-6811.2006.00113.x
40. Ehrilich, K. B., Cassidy, J., & Dykas, M. J. (2011). Reporter discrepancies among parents, adolescents, and peers: Adolescent attachment and informant depressive symptoms as explanatory factors. *Child Development, 82,* 999-1012. https://doi.org/10.1111/j.1467-8624.2010.01530.x
41. Engels, R., Dinkenauer, C., Meeus, W., & Dekovič, M. (2000). Hechting aan ouders en welbevinden van adolescenten: de invloed van sociale vaardigheden en sociale competentie. *Pedagogiek, 4,* 1567-7109*.*
42. Essau, C. A. (2004). The association between family factors and depressive disorders in adolescents. *Journal of Youth and Adolescence, 33,* 365-372. https://doi.org/10.1023/b:joyo.0000037630.01098.d4
43. Formoso, D., Gonzales, N. A., & Aiken, L. S. (2000). Family conflict and children's internalizing and externalizing behavior: Protective factors. *American Journal of Community Psychology*, *28*, 175-199. https://doi.org/10.1023/a:1005135217449
44. Fox, M. K., & Borelli, J. L. (2015). Attachment Moderates the Association Between Mother and Child Depressive Symptoms. *Psi Chi Journal of Psychological Research*, *20*.
45. Gatz, A. O. (2000). *The reliability and validity of the" Parent Attachment Scale": A measure of adolescent perceptions of parental attachment behaviors* (Doctoral dissertation). Retrieved from ProQuest Dissertations and Theses.
46. Gaylord-Harden, N. K., Taylor, J. J., Campbell, C. L., Kesselring, C. M., & Grant, K. E. (2009). Maternal attachment and depressive symptoms in urban adolescents: The influence of coping strategies and gender. *Journal of Clinical Child & Adolescent Psychology*, *38*, 684-695. https://doi.org/10.1080/15374410903103569
47. Glazebrook, K., Townsend, E., & Sayal, K. (2015). The Role of Attachment Style in Predicting Repetition of Adolescent Self‐Harm: A Longitudinal Study. *Suicide and life-threatening behavior*, *45*, 664-678. https://doi.org/10.1111/sltb.12159
48. Goodman, G., Stroh, M., & Valdez, A. (2012). Do attachment representations predict depression and anxiety in psychiatrically hospitalized prepubertal children? *Bulletin of the Menninger Clinic*, *76*, 260-289. https://doi.org/10.1521/bumc.2012.76.3.260
49. Graham, C. A., & Easterbrooks, M. A. (2000). School-aged children’s vulnerability to depressive symptomatology: The role of attachment security, maternal depressive sympatomatology, and economic risk. *Developmental Psychopathology, 12,* 201-213. https://doi.org/10.1017/s0954579400002054
50. Gullone, E., Ollendick, T. H., & King, N. J. (2006). The role of attachment representation in the relationship between depressive symptomatology and social withdrawal in middle childhood. *Journal of Child and Family Studies*, *15*, 263-277. https://doi.org/10.1007/s10826-006-9034-0
51. Halamová, M., & Popelková, M. (2015). Attachment representation as predictor of internalizing problems in middle childhood. *Psychology and its Contexts*, *6*, 49-63.
52. Halamova, M., & Popelkova, M. (2015). Mother-child attachment security and symptoms of depression in middle childhood. *Ad Alta: Journal of Interdisciplinary Research*, *5*.
53. Henderson, S. (2009). *Attachment security as a predictor of blood glucose control in adolescents with Type 1 Diabetes, when the roles of additional psychological factors are considered* (Master’s thesis, the University of Edinburgh). Retrieved from https://www.era.lib.ed.ac.uk/handle/1842/4915
54. Hüsler, G., Blakeney, R., & Werlen, E. (2005). Adolescent risk: The co-occurrence of illness, suicidality, and substance use. *Journal of Youth and Adolescence*, *34*, 547. https://doi.org/10.1007/s10964-005-8945-1
55. Irons, C., & Gilbert, P. (2005). Evolved mechanisms in adolescent anxiety and depression symptoms: The role of the attachment and social rank systems. *Journal of Adolescence*, *28*, 325-341. https://doi.org/10.1016/j.adolescence.2004.07.004
56. Jinyao, Y., Xiongzhao, Z., Auerbach, R. P., Gardiner, C. K., Lin, C., Yuping, W., & Shuqiao, Y. (2012). Insecure attachment as a predictor of depressive and anxious symptomology. *Depression and anxiety*, *29*, 789-796. https://doi.org/10.1002/da.21953
57. Kamkar, K., Doyle, A. B., & Markiewicz, D. (2012). Insecure attachment to parents and depressive symptoms in early adolescence: Mediating roles of attributions and self-esteem. *International Journal of Psychological Studies*, *4*, 3. https://doi.org/10.5539/ijps.v4n2p3
58. Katz, J., Petracca, M., & Rabinowitz, J. (2009). A retrospective study of daughters’ emotional role reversal with parents, attachment anxiety, excessive reassurance-seeking, and depressive symptoms. *The American Journal of Family Therapy, 37,* 185-195. https://doi.org/10.1080/01926180802405596
59. Kenny, M. E., Lomax, R., Brabeck, M., & Fife, J. (1998). Longitudinal pathways linking adolescent reports of maternal and paternal attachments to psychological well-being. *The Journal of Early Adolescence*, *18*, 221-243. https://doi.org/10.1177/0272431698018003001
60. Kenny, M. E., Gallagher, L. A., Alvarez-Salvat, R., & Silsby, J. (2002). Sources of support and psychological distress among academically successful inner-city youth. *Adolescence*, *37*, 161.
61. Kerns, K. A., Brumariu, L. E., & Seibert, A. (2011). Multi-method assessment of mother-child attachment: Links to parenting and child depressive symptoms in middle childhood. *Attachment and Human Development, 13,* 315-333. https://doi.org/10.1080/14616734.2011.584398
62. Kerr, M. A. (2010). *Depressed mothers and problem behaviours in their adolescent daughters: The mediating roles of parenting and attachment security* (Doctoral dissertation. Canada: University of Ottawa.
63. Kim, S. Y., Hou, Y., & Gonzalez, Y. (2017). Language brokering and depressive symptoms in mexican‐american adolescents: Parent–child alienation and resilience as moderators. *Child development*, *88*, 867-881. https://doi.org/10.1111/cdev.12620
64. Korbel, C. D. (2009). *Maternal attachment: Associations with diabetes and psychosocial adjustment during adolescence* (Doctoral dissertation). Retrieved from ProQuest Dissertations and Theses.
65. Kullik, A., & Petermann, F. (2013). Attachment to parents and peers as a risk factor for adolescent depressive disorders: The mediating role of emotion regulation. *Child Psychiatry & Human Development*, *44*, 537-548. https://doi.org/10.1007/s10578-012-0347-5
66. Laible, D. J., Carlo, G., & Raffaelli, M. (2000). The differential relations of parent and peer attachment to adolescent adjustment. *Journal of Youth and Adolescence, 29,* 45–59.
67. Leas, L., & Mellor, D. (2000). Prediction of delinquency: The role of depression, risk-taking, and parental attachment. *Behaviour Change, 17,* 155-166. https://doi.org/10.1375/bech.17.3.155
68. Lee, A., & Hankin, B. (2009). Insecure attachment, dysfunctional attitudes, and low self-esteem predicting prospective symptoms of depression and anxiety during adolescence. *Journal of Clinical Child and Adolescence Psychology, 38,* 219-231. https://doi.org/10.1080/15374410802698396
69. Leenaars, L. S., Dane, A. V., & Marini, Z. A. (2008). Evolutionary perspective on indirect victimization in adolescence: The role of attractiveness, dating and sexual behavior. *Aggressive Behavior*, *34*, 404-415. https://doi.org/10.1002/ab.20252
70. Lecompte, V., Moss, E., Cyr, C., & Pascuzzo, K. (2014). Preschool attachment, self-esteem and the development of preadolescent anxiety and depressive symptoms. *Attachment & human development*, *16*, 242-260. https://doi.org/10.1080/14616734.2013.873816
71. Li, S. T., Albert, A. B., & Dwelle, D. G. (2014). Parental and peer support as predictors of depression and self-esteem among college students. *Journal of college student development*, *55*, 120-138. https://doi.org/10.1353/csd.2014.0015
72. Li, J. B., Delvecchio, E., Lis, A., Nie, Y. G., & Di Riso, D. (2015). Parental attachment, self-control, and depressive symptoms in Chinese and Italian adolescents: Test of a mediation model. *Journal of adolescence*, *43*, 159-170. https://doi.org/10.1016/j.adolescence.2015.06.006
73. Liebman, S. E. (1997). Familial predictors of depressed mood in adolescence. *Dissertation Abstracts International: Section B: The Sciences and Engineering, 59,* 2-877.
74. Liu, Y. (2006). Paternal/maternal attachment, peer support, social expectations of peer interaction, and depressive symptoms. *Adolescence, 41,* 705-721.
75. Marini, Z. A., Dane, A. V., Bosacki, S. L., & Ylc-Cura. (2006). Direct and indirect bully-victims: Differential psychosocial risk factors associated with adolescents involved in bullying and victimization. *Aggressive Behavior, 32,* 551-569. https://doi.org/10.1002/ab.20155
76. Marsh, P., McFarland, F. C., Allen, J. P., Mcelhaney, K. B., & Land, D. (2003). Attachment, autonomy, and multifinality in adolescent internalizing and risky behavioral symptoms. *Developmental Psychopathology, 15,* 451-467. https://doi.org/10.1017/s0954579403000245
77. McCann, M., Higgins, K., Perra, O., McCartan, C., & McLaughlin, A. (2014). Adolescent ecstasy use and depression: cause and effect, or two outcomes of home environment? *The European Journal of Public Health*, *24*, 845-850. https://doi.org/10.1093/eurpub/cku062
78. McConnell, M. (2008). *Attachment, depression, and perceptions of parenting among adolescent mothers* (Master’s thesis, San Jose State University). Retrieved from http://scholarworks.sjsu.edu/cgi/viewcontent.cgi?article=4533&context=etd_theses
79. Milan, S., Snow, S., & Belay, S. (2009). Depressive symptoms in mothers and children: Preschool attachment as a moderator of risk. *Developmental Psychology, 45,* 1019-1033. https://doi.org/10.1037/a0016164
80. Milne, L. C., & Lancaster, S. (2001). Predictors of depression in female adolescents. *Adolescence*, *36*, 207.
81. Moss, E., Smolla, N., Cyr, C., Dubois-Comtois, K., Mazzarello, T., & Berthiaume, C. (2006). Attachment and behavior problems in middle childhood as reported by adult and child informants. *Development and psychopathology*, *18*, 425-444. https://doi.org/10.1017/s0954579406060238
82. Moutsiana, C., Fearon, P., Murray, L., Cooper, P., Goodyer, I., Johnstone, T., & Halligan, S. (2014). Making an effort to feel positive: insecure attachment in infancy predicts the neural underpinnings of emotion regulation in adulthood. *Journal of Child Psychology and Psychiatry*, *55*, 999-1008. https://doi.org/10.1111/jcpp.12198
83. Muris, P., Meesters, C., & Van den Berg, S. (2003). Internalizing and externalizing problems as correlates of self-reported attachment style and perceived parental rearing in normal adolescents. *Journal of Child and Family Studies, 12,* 171–183. https://doi.org/10.1016/s0022-3999(03)00616-0
84. Nicholas, C. (1997). *Adolescents' perceived attachment to parents and its relationship to depression* (Doctoral dissertation)*.* Retrieved from ProQuest Dissertations and Theses.
85. Noom, M. J., Deković, M., & Meeus, W. H. (1999). Autonomy, attachment and psychosocial adjustment during adolescence: A double-edged sword? *Journal of adolescence*, *22*, 771-783. https://doi.org/10.1006/jado.1999.0269
86. Omidvar, B., Bahrami, F., Fatehizade, M., Etemadi, O., & Ghanizadeh, A. (2014). Attachment quality and depression in Iranian adolescents. *Psychological Studies*, *59*, 309-315. https://doi.org/10.1007/s12646-014-0250-1
87. O'Shea, G., Spence, S. H., & Donovan, C. L. (2014). Interpersonal factors associated with depression in adolescents: Are these consistent with theories underpinning interpersonal psychotherapy? *Clinical psychology & psychotherapy*, *21*, 548-558. https://doi.org/10.1002/cpp.1849
88. Pan, Y., Zhang, D., Liu, Y., Ran, G., & Teng, Z. (2016). Different effects of paternal and maternal attachment on psychological health among Chinese secondary school students. *Journal of Child and Family Studies*, *25*, 2998-3008. https://doi.org/10.1007/s10826-016-0463-0
89. Papafratzeskakou, E., Kim, J., Longo, G. S., & Riser, D. K. (2011). Peer victimization and depressive symptoms: Role of peers and parent–child relationship. *Journal of Aggression, Maltreatment & Trauma*, *20*, 784-799. https://doi.org/10.1080/10926771.2011.608220
90. Papini, D. R., Roggman, L. A., & Anderson, J. (1991). Early-adolescent perceptions of attachment to mother and father a test of the emotional-distancing and buffering hypotheses. *The Journal of Early Adolescence, 11,* 258-275. https://doi.org/10.1177/0272431691112006
91. Papini, D. R., & Roggman, L. A. (1992). Adolescent perceived attachment to parents in relation to competence, depression, and anxiety: A Longitudinal Study. *The Journal of Early Adolescence, 12,* 420-440. https://doi.org/10.1177/0272431692012004005
92. Pettineo, L.P. (2011), *Stress Generation in Urban African American Girls: The Role of Mother-Daughter Attachment and Maternal Acceptance* (master’s thesis). Retrieved from ProQuest Dissertations and Theses.
93. Priddis, L., & Howieson, N. (2012). Insecure attachment patterns at five years. What do they tell us? *Early Child Development and Care, 182,* 45-58. https://doi.org/10.1080/03004430.2010.537334
94. Rawatlal, N., Kliewer, W., & Pillay, B. J. (2015). Adolescent attachment, family functioning and depressive symptoms. *South African Journal of Psychiatry*, *21*, 80-85. https://doi.org/10.7196/sajp.8252
95. Rezvan, S., Bahrami, F., Abedi, M., MacLeod, C., Doost, H. T. N., & Ghasemi, V. (2012). Attachment insecurity as a predictor of obsessive–compulsive symptoms in female children. *Counselling Psychology Quarterly*, *25*, 403-415. https://doi.org/10.1080/09515070.2012.736156
96. Ridenour, T. A., Greenberg, M. T., & Cook, E. T. (2006). Structure and validity of people in my life: A self-report measure of attachment in late childhood. *Journal of Youth and Adolescence*, *35*, 1037-1053. https://doi.org/10.1007/s10964-006-9070-5
97. Roalson, L. A. (2007). *The unique and moderating effects of religious, family and school connectedness on early adolescent adjustment* (Doctoral dissertation). Retrieved from https://repositories.lib.utexas.edu/handle/2152/3284
98. Roelofs, J., Chris, L., Ruijten, T., & Lobbestael, J. (2011). The mediating role of early maladaptive schemas in the relation between quality of attachment relationships and symptoms of depression in adolescents. *Behavioural and Cognitive Psychotherapy, 39,* 471-479. https://doi.org/10.1017/s1352465811000117
99. Roelofs, J., Meesters, C., Ter Huurne, M., Bamelis, L., & Muris, P. (2006). On the links between attachment style, parental rearing behaviours, and internalizing and externalizing problems in non-clinical children. *Journal of Child and Family Studies, 15,* 331-344. https://doi.org/10.1007/s10826-006-9025-1
100. Ruijten, T., Roelofs, J., & Rood, L. (2011). The mediating role of rumination in the relation between quality of attachment relations and depressive symptoms in non-clinical adolescents. *Journal of Child and Family Studies, 20,* 452-459. https://doi.org/10.1007/s10826-010-9412-5
101. Salzman, J. P. (1996). Primary attachment in female adolescents: Association with depression, self-esteem, and maternal identification. *Psychiatry: Interpersonal and  Biological Processes, 59,* 20-33. https://doi.org/10.1080/00332747.1996.11024748
102. Schoenfelder, E. N., Sandler, I. N., Wolchik, S., & MacKinnon, D. (2011). Quality of social relationships and the development of depression in parentally-bereaved youth. *Journal of Youth and Adolescence, 40,* 85-96. https://doi.org/10.1007/s10964-009-9503-z
103. Shochet, I. M., Homel, R., Cockshaw, W. D., & Montgomery, D. T. (2008). How do school connectedness and attachment to parents interrelate in predicting adolescents depressive symptoms? *Journal of Clinical Child and Adolescent Psychology, 37,* 676-681. https://doi.org/10.1080/15374410802148053
104. Silverman, A. (2003). *Social support as a mediator of attachment style and depression in adolescents* (Doctoral dissertation). Retrieved from https://scholar.uwindsor.ca/cgi/viewcontent.cgi?article=8199&context=etd
105. Sim, T. N., & Yow, A. S. (2011). God attachment, mother attachment, and father  attachment in early and middle adolescence. *Journal of Religion and Health, 50,* 264-278. https://doi.org/10.1007/s10943-010-9342-y
106. Smojver-Ažić, S., Martinac Dorčić, T., & Živčić-Bećirević, I. (2015). Attachment to Parents and Depressive Symptoms in College Students: The Mediating Role of Initial Emotional Adjustment and Psychological Needs. *Psihologijske teme*, *24*, 135-153.
107. Speekenbrink, S., van Hoof, M., van Lang, N., & Vermeien, R. (2016). Emotieregulatie en gehechtheidsrepresentaties in adolescenten met een klinische depressie. *Gedragstherapie*, 49, 98-121.
108. Stebbins, P.A. (2004). *Adolescent depression, vulnerability factors for boys and girls* (Doctoral dissertation). Retrieved from ProQuest Dissertations and Theses.
109. Sund, A. M., & Wichstrøm, L. (2002). Insecure attachment as a risk factor for future depressive symptoms in early adolescence. *Journal of the American Academy of Child and Adolescents Psychiatry, 41,* 1478-1485. https://doi.org/10.1097/00004583-200212000-00020
110. Suzuki, H., & Tomoda, A. (2015). Roles of attachment and self-esteem: impact of early life stress on depressive symptoms among Japanese institutionalized children. *BMC psychiatry*, *15*, 8. https://doi.org/10.1186/s12888-015-0385-1
111. Szalai, T. D., Czeglédi, E., Vargha, A., & Grezsa, F. (2017). Parental Attachment and Body Satisfaction in Adolescents. *Journal of Child and Family Studies*, *26*, 1007-1017.
112. Trapani, J. N. (2007). *The differential influence of multiple reporters on the relationship between attachment in infancy and internalizing behaviors in middle childhood* (Doctoral dissertation). Retrieved from https://repositories.lib.utexas.edu/handle/2152/2974
113. Van de Walle, M., Bijttebier, P., Braet, C., & Bosmans, G. (2016). Attachment anxiety and depressive symptoms in middle childhood: The role of repetitive thinking about negative affect and about mother. *Journal of Psychopathology and Behavioral Assessment*, *38*, 615-630. https://doi.org/10.1007/s10862-016-9552-z
114. Van Hoof, M. J., van Lang, N. D., Speekenbrink, S., van IJzendoorn, M. H., & Vermeiren, R. R. (2015). Adult Attachment Interview differentiates adolescents with Childhood Sexual Abuse from those with clinical depression and non-clinical controls. *Attachment & human development*, *17*, 354-375. https://doi.org/10.1080/14616734.2015.1050420
115. Van Leeuwen, N., Rodgers, R., Régner, I., & Chabrol, H. (2010). The role of acculturation in suicidal ideation among second-generation immigrant adolescents in France. *Transcultural psychiatry*, *47*, 812-832. https://doi.org/10.1177/1363461510382154
116. Vassallo, S., Edwards, B., Renda, J., & Olsson, C. A. (2014). Bullying in early adolescence and antisocial behavior and depression six years later: What are the protective factors? *Journal of school violence*, *13*, 100-124. https://doi.org/10.1080/15388220.2013.840643
117. Venta, A., Shmueli-Goetz, Y., & Sharp, C. (2014). Assessing attachment in adolescence: A psychometric study of the Child Attachment Interview. *Psychological Assessment*, *26*, 238. https://doi.org/10.1037/a0034712
118. Venta, A., Mellick, W., Schatte, D., & Sharp, C. (2014). Preliminary evidence that thoughts of thwarted belongingness mediate the relations between level of attachment insecurity and depression and suicide-related thoughts in inpatient adolescents. *Journal of Social and Clinical Psychology*, *33*, 428-447. https://doi.org/10.1521/jscp.2014.33.5.428
119. Vivona, J. M. (2000). Parental attachment styles of late adolescents: Qualities of attachment relationships and consequences for adjustment. *Journal of Counseling Psychology, 47,* 316-329. https://doi.org/10.1037//0022-0167.47.3.316
120. Wilkinson, R. B. (2010). Best friend attachment versus peer attachment in the prediction of adolescent psychological adjustment. *Journal of Adolescence*, *33*, 709-717. https://doi.org/10.1016/j.adolescence.2009.10.013
121. Wong, S. L. (2000). Depression level in inner-city Asian American adolescents: The contributions of cultural orientation and interpersonal relationships. *Journal of Human Behavior in the Social Environment, 3,* 49-64. https://doi.org/10.1300/j137v03n03_05
122. Woodhouse, S. S., Ramos-Marcuse, F., Ehrlich, K. B., Warner, S., & Cassidy, J. (2010). The role of adolescent attachment in moderating and mediating the links between parent and adolescent psychological symptoms. *Journal of Clinical Child and Adolescents Psychology, 39,* 51-63. https://doi.org/10.1080/15374410903401096
123. Yeh, C. J., Liao, H. Y., Ma, P. W. W., Shea, M., Okubo, Y., Kim, A. B., & Atkins, M. S. (2014). Ecological risk and protective factors of depressive and anxiety symptoms among low-income, Chinese immigrant youth. *Asian American Journal of Psychology*, *5*, 190.
     **Appendix C**

**Characteristics of included studies**

| **Authors** | **Year** | ***N*** | **Study design** | **Age Att** | **Age Dep** | **Att measure** | **Att figure** | **Dep measure** |
| --- | --- | --- | --- | --- | --- | --- | --- | --- |
| 1. Abela et al | 2005 | 140 | Cross | 9,80 | 9,80 | IPPA | General | CDI, K-SADS, BDI |
| 1. Agerup et al | 2015 | 159 | Long | 15,00 | 20,00 | IPPA | Mother  Father | K-SADS-PL |
| 1. Aifuwa | 2016 | 75 | Cross | n/a | n/a | PAQ | Parent | CES-D |
| 1. Allen et al | 2007 | 167 | Long | 13,36  14,29  15,22 | 13,36  14,29  15,22 | AAI Q-set | Parent | CDI |
| 1. Armsden et al | 1990 | 55 | Cross | 13,53 | 13,53 | IPPA | Parent | K-SADS, CDI |
| 1. Armsden et al | 1987 | 86 | Cross | 18,60 | 18,60 | IPPA | Parent | ASI |
| 1. Bamaca-Colbert | 2012 | 170 | Cross | 12,26  15,20 | 12,26  15,20 | IPPA | Mother | CES-D |
| 1. Bauman et al | 2006 | 50 | Cross | 12,40  13,00 | 12,40  13,00 | IPPA | Mother | CDI |
| 1. Bennett et al | 2005 | 80 | Long | 1,13 | 7,50 | SSP | Mother | BASC |
| 1. Bogard | 2005 | 201 | Cross | 12,60 | 12,60 | IPPA | Mother  Father | CDI |
| 1. Borelli et al | 2010 | 97 | Long | 10,10 | 10,10 | CAI | Parent | HIF, PANAS-C |
| 1. Borelli et al | 2017 | 106 | Long | 10,30 | 10,30 | SS | Mother | CDI |
| 1. Bosmans et al | 2016 | 60 | Cross | 10,45 | 10,45 | ECR-RC | Mother | CES-D |
| 1. Bosquet | 2006 | 155 | Long | 1,00 | 17,50 | SSP | Mother | K-SADS |
| 1. Branje et al | 2010 | 1313 | Long | n/a | n/a | IPPA | Mother  Father | CDI |
| 1. Brenning et al | 2011 | 303 | Cross | 12,00 | 12,00 | ECR-RC | Mother | CDI |
| 1. Brenning et al | 2012 | 238 | Cross | 14,46 | 14,46 | ECR-RC | Mother | CDI |
| 1. Brenning et al | 2012 | 339 | Cross | 12,60 | 12,60 | ECR-RC | Mother | CDI |
| 1. Brenning et al | 2013 | 289 | Long | 12,00  13,00  14,00  15,00 | 12,00  13,00  14,00  15,00 | ECR-RC | Mother | CDI |
| 1. Brodie | 2004 | 169 | Cross | n/a | n/a | IPPA | Mother  Father | BDI |
| 1. Buist et al | 2004 | 285 | Long | 13,50  14,50  15,50 | 13,50  14,50  15,50 | IPPA | Parent | NPBL |
| 1. Bureau et al | 2009 | 47 | Long | 1,50 | 8,00  19,00 | SSP | Mother | DDPCA, CES-D |
| 1. Burge et al | 1997 | 137 | Long | 18,17 | 18,17 | IPPA  RAAS | Parent  General | SCID |
| 1. Cawthorpe et al | 2004 | 73 | Cross | 15,50 | 15,50 | AAQ | Parent | DISC-R |
| 1. Chaodiang | 2008 | 950 | Cross | 17,00 | 17,00 | IPPA | Mother  Father | CES-D |
| 1. Chedebois et al | 2009 | 292 | Cross | 17,00 | 17,00 | IPPA | Parent | CES-D |
| 1. Chesmore | 2017 | 493 | Cross | 10,40 | 10,40 | IPPA | Parent | TSCC |
| 1. Cole-Detke et al | 1996 | 41 | Cross | 18,60 | 18,60 | AAI | Parent | BDI |
| 1. Constantine et al | 2006 | 283 | Cross | 16,57 | 16,57 | IPPA | Parent | CDI |
| 1. Cooper-Newark | 2015 | 180 | Cross | 16,69 | 16,69 | ECR-RC GSF | General | BDI |
| 1. Cotterell | 1992 | 29 | Cross | 15,90 | 15,90 | IPPA | Parent | BDI |
| 1. Crittenden et al | 2015 | 44 | Both | 5,70 | 5,70  6,20 | PAA | Parent | CDI |
| 1. Demidenko | 2015 | 116 | Cross | n/a | n/a | IPPA | Father | Diagnosis in clinic (file information) |
| 1. De Minzi | 2006 | 1019 | Cross | 10,06 | 10,06 | KSS | Parent  Mother  Father | DDPCA |
| 1. Dhillon & Kanwar | 2015 | 100 | Cross | 13,25  13,94 | 13,25  13,94 | IPPA | Mother  Father | CES-D |
| 1. Diamond et al | 2002 | 15 | Long | 14,90 | 14,90 | IPPA | Mother | BDI |
| 1. Djuardin et al | 2016 | 98 | Both | 10,39  11,98 | 10,39  11,98 | ECR-RC | Mother | CES-D |
| 1. Duchesne et al | 2014 | 416 | Long | 11,00 | 11,00  12,00  13,00  14,00  15,00  16,00 | SS | Mother  Father | CDI-S |
| 1. Eberhart & Hammen | 2006 | 97 | Cross | 17,92 | 17,92 | IPPA  RAAS | Parent  General | BDI |
| 1. Ehrlich et al | 2011 | 189 | Cross | 16,50 | 16,50 | AAI | Parent | CDI, CES-D |
| 1. Engels et al | 2000 | 508 | Cross | 13,00  16,50 | 13,00  16,50 | IPPA | Parent | DML |
| 1. Essau | 2004 | 600 | Cross | 14,30 | 14,30 | IPPA | Parent | CIDI |
| 1. Formoso et al | 2000 | 284 | Cross | 13,30 | 13,30 | IPPA | Mother  Father | CDI |
| 1. Fox & Borelli | 2015 | 107 | Cross | 9,74 | 9,74 | CAI | Mother | CDI |
| 1. Gatz | 2000 | 162 | Cross | n/a | n/a | PAS | Mother  Father | BDI |
| 1. Gaylord-Harden et al | 2009 | 392 | Both | 12,03 | 12,03  13,03 | IPPA | Mother | CDI |
| 1. Glazebrook et al | 2015 | 51 | Cross | n/a | n/a | CAI | Mother | HADS |
| 1. Goodman et al | 2012 | 36 | Cross | 7,97 | 7,97 | ASCT, ADP | Parent | CDI, DICA |
| 1. Graham et al | 2000 | 65 | Cross | 8,00 | 8,00 | MC | Mother | DDPCA |
| 1. Gullone et al | 2006 | 326 | Cross | 9,02 | 9,02 | FDP | General | CDI |
| 1. Halamova & Poplkova | 2015 | 139 | Cross | 10,79 | 10,79 | SS | Parent | CDI |
| 1. Halamova & Poplkova | 2015 | 151 | Cross | 11,20 | 11,20 | SS | Mother | CDI |
| 1. Henderson | 2009 | 86 | Cross | 16,10 | 16,10 | A-RQ | General | HADS Depression |
| 1. Hüsler et al | 2005 | 1028 | Cross | n/a | n/a | IPPA | Parent | CES-D |
| 1. Irons & Gilbert | 2005 | 140 | Cross | 14,63 | 14,63 | RQ | General | CDI |
| 1. Jinyao et al | 2012 | 662 | Cross | 20,11 | 20,11 | ASQ | General | MASQ |
| 1. Kamkar et al | 2012 | 140 | Cross | 12,65 | 12,65 | ARSQ | Mother  Father | CDI |
| 1. Katz et al | 2009 | 163 | Cross | n/a | n/a | AAS | General | CES-D |
| 1. Kenny et al | 1998 | 132 | Both | 14,00  15,00 | 14,00  15,00 | PAQ | Father  Mother | CDI |
| 1. Kenny et al | 2002 | 100 | Cross | 16,00 | 16,00 | PAQ | Mother  Father | CDI |
| 1. Kerns et al | 2011 | 87 | Cross | 11,32 | 11,32 | SST, SS | Mother | CDI |
| 1. Kerr | 2010 | 151 | Both | 11,60 | 11,60  12,60 | IPPA | Mother | BDI-II |
| 1. Kim et al | 2017 | 557 | Cross | 12,96 | 12,96 | IPPA | Parent | CES-D |
| 1. Korbel | 2009 | 100 | Cross | 13,46 | 13,46 | ECR, SS | Mother | CDI |
| 1. Kullik & Peterman | 2013 | 248 | Cross | 14,41 | 14,41 | IPPA | Parent | CES-D |
| 1. Laible et al | 2000 | 89 | Cross | 16,00 | 16,00 | IPPA | Parent | CDI |
| 1. Leas et al | 2000 | 108 | Cross | 18,90 | 18,90 | IPPA | Parent | BDI |
| 1. Lee et al | 2009 | 350 | Long | 14,50 | 14,50  14,81 | ECR-R | General | CDI |
| 1. Leenaars et al | 2008 | 2319 | Cross | n/a | n/a | IPPA | Parent | CES-D |
| 1. Lecompte et al | 2014 | 58 | Long | 3,70 | 11,70 | SSP | Mother | DIQ |
| 1. Li et al | 2014 | 197 | Cross | 18,38 | 18,38 | IPPA | Parent  Mother | BDI-II |
| 1. Li et al | 2015 | 2632 | Cross | 13,84  13,90 | 13,84  13,90 | IPPA-R | Mother  Father | CDI |
| 1. Liebman | 1997 | 125 | Long | 16,00  18,00 | 16,00  18,00 | AAI Q set, IPPA | Parent  Mother  Father | BDI, YSR, WAI |
| 1. Liu | 2006 | 1289 | Cross | 14,00 | 14,00 | CPS | Mother  Father | CDI |
| 1. Marini et al | 2006 | 7290 | Cross | 15,58 | 15,58 | IPPA | Mother | CES-D |
| 1. Marsh et al | 2003 | 123 | Cross | 15,90 | 15,90 | AAI Q set | Parent | BDI |
| 1. McCann et al | 2014 | 5371 | Long | 14,20 | 16,71 | IPPA | Parent | CDI |
| 1. McConnell | 2008 | 27 | Cross | 17,10 | 17,10 | AAQ, AUAQ | General | BDI |
| 1. Milan et al | 2009 | 938 | Long | 3,00 | 11,00 | SSP | Mother | CDI |
| 1. Milne & Lancaster | 2001 | 59 | Cross | 17,70 | 15,70 | IPPA | Parent | DEQ-A |
| 1. Moss et al | 2006 | 72 | Long | 6,30 | 8,50 | MC | Mother | Dominic Test |
| 1. Moutisiana et al | 2014 | 54 | Long | 1,50 | 22,00 | SSP | Mother | KSAD, SCID, CES-D |
| 1. Muris et al | 2001 | 155 | Cross | 12,90 | 12,90 | IPPA | Parent | CDI |
| 1. Nicholas | 1997 | 166  188 | Cross | 15,95  16,08 | 15,95  16,08 | IPPA | Mother  Father | CES-D |
| 1. Noom et al | 2000 | 400 | Cross | 15,00 | 15,00 | IPPA | Mother  Father | DML |
| 1. Omidvar et al | 2014 | 395 | Cross | 15,70 | 15,70 | IPPA | Mother  Father | BDI |
| 1. O’Shea et al | 2013 | 61 | Cross | 15,19 | 15,19 | ASQ | General | K-SADS-E |
| 1. Pan | 2016 | 1506 | Cross | 15,21 | 15,21 | IPPA | Mother  Father | CES-D |
| 1. Papafratzeskakou | 2011 | 261 | Cross | 12,50 | 12,50 | IPPA | Parent | CDI |
| 1. Papini et al | 1991 | 231 | Cross | 12,80 | 12,80 | IPPA | Mother  Father | CDI |
| 1. Papini et al | 1992 | 47 | Long | 12,60  13,18  13,76 | 12,60  13,18  13,76 | IPPA | Mother  Father | CDI |
| 1. Pettineo | 2011 | 177 | Both | 14,00 | 14,00  15,00 | IPPA | Parent | YSR |
| 1. Priddis et al | 2012 | 83 | Long | 5,00 | 12,00 | SSP | Mother | CDI |
| 1. Rawatlal et al | 2015 | 206 | Cross | 13,02 | 13,02 | ECR | Parent | CDI |
| 1. Rezvan et al | 2012 | 221 | Cross | 11,00 | 11,00 | IPPA | Parent | DSRS |
| 1. Roalson | 2007 | 167 | Cross | 12,47 | 12,47 | IPPA | Parent | CES-DC |
| 1. Ridenour et al | 2006 | 310 | Cross | 11,02 | 11,02 | PIML | Parent | RCDS |
| 1. Roelofs et al | 2006 | 230 | Cross | 10,50 | 10,50 | RQ | Mother  Father | RCADS |
| 1. Roelofs et al | 2011 | 222 | Cross | 14,70 | 14,70 | IPPA | Parent | BDI |
| 1. Ruijten et al | 2011 | 455 | Cross | 14,30 | 14,30 | IPPA | Parent | BDI |
| 1. Salzman | 1996 | 21 | Cross | n/a | n/a | AAI | General | CES-D  Interview depression |
| 1. Schoenfelder et al | 2011 | 109 | Long | 17,30 | 11,30  11,30 | IPPA | Parent | CBCL  YSR |
| 1. Shochet et al | 2008 | 152 | Cross | 15,20 | 15,20 | PAQ | Parent | CDI |
| 1. Silverman | 2003 | 451 | Cross | 16,02 | 16,02 | RSQ, RQ | General | BDI, CES-D |
| 1. Sim et al | 2011 | 130 | Cross | 12,58  15,50 | 12,58  15,50 | Sim & Loh | Mother  Father | AADS |
| 1. Smojver Azic | 2015 | 219 | Cross | 19,02 | 21,02 | ECR | Parent | BDI-II |
| 1. Speekenbrink et al | 2016 | 50 | Cross | 15,88 | 15,88 | AAI | General | Diagnosis by clinical psychologist |
| 1. Stebbins | 2008 | 510 | Cross | 12,89  13,68  14,60 | 12,89  13,68  14,60 | AAQ | Parent | CDI |
| 1. Sund et al | 2002 | 1973 | Long | 13,70 | 13,70 | IPPA | Parent | MFQ |
| 1. Suzuki & Tomoda | 2015 | 342 | Cross | 13,50 | 13,50 | IWMQ | General | BDSSC |
| 1. Szalai et al | 2017 | 5214 | Cross | 14,80 | 14,80 | ECR-RC | Mother  Father | CDI |
| 1. Trapani | 2007 | 55 | Long | 1,13 | 7,00 | SSP | Mother | BPI |
| 1. Van de Wallen et al | 2016 | 381 | Cross | 10,91  11,25 | 10,91  11,25 | ECR-RC | Mother | CDI |
| 1. Van Hoof et al | 2015 | 56 | Cross | 15,54 | 15,54 | AAI | General | CDI, ADIS |
| 1. Van Leeuwen | 2010 | 292 | Cross | 17,10 | 17,10 | IPPA | Parent | CES-D |
| 1. Vassallo et al | 2014 | 993 | Long | 13,50 | 19,50 | IPPA | Parent | DASS21 |
| 1. Venta et al | 2014 | 114 | Cross | 14,68 | 14,68 | SS | Mother | BDI, YSR |
| 1. Venta et al | 2014 | 194 | Cross | 15,97 | 15,97 | CAI | Parent  Mother  Father | YSR, CBCL |
| 1. Vivona | 2000 | 77 | Cross | 19,98 | 19,98 | IPPA | Parent | BDI |
| 1. Wilkinson | 2010 | 495 | Cross | 16,41 | 16,41 | IPPA | Mother  Father | Wilkinson Scale for Depression |
| 1. Wong | 2000 | 144 | Cross | 15,70 | 15,70 | IPPA | Parent | CES-D |
| 1. Woodhouse et al | 2010 | 189 | Cross | n/a | n/a | AAI  PSBS | Parent  Mother  Father | CDI |
| 1. Yeh et al | 2013 | 284 | Cross | 19,02 | 19,02 | IPPA | Parent  Mother  Father | CDI |

Note. *N* = *N* total sample; Long = longitudinal study; Cross = cross-sectional study; Age att = age at attachment measurement; Age dep = age at depression measurement; Att measure = attachment measure; Att figure = attachment figure; Dep measure = depression measure.

Attachment measures: AAI = Adult Attachment Interview; AAI-Q sort = Adult Attachment Interview Q sort; AAQ = Adolescent Attachment Questionaire; ADP = Attachment Doll Play; ASCT = Attachment Story-Completion Task; A-RQ = Adolescent Relationships Scale; ARSQ = Adolescent Relationship Scales Questionnaire; ASQ = Attachment Styles Questionnaire; AUAQ = Adolescent Unresolved Attachment Questionnaire; CAI = Child Attachment Interview; CPS = Child’s Perception of Security; ECR-R = Experiences in Close Relationships-Revised Questionnaire; FDP = Family Drawing Procedure; IPPA = Inventory of Parent and Peer Attachment; IWMQ = Internal Working Models Questionnaire; MC = Main and Cassidy procedure; PAA = Preschool Assessment of Attachment; PAS = Parent Attachment Scale; PSBS = Parents as Secure Base Scale; PAQ = Parental Attachment Questionnaire; PIML = People In My Life; RAAS = Revised Adult Attachment Scale; RQ = Relationship Questionnaire; SS = Security Scale; SSP = Strange Situation Procedure; SST = Story Stam Task.

Depression measures: ADIS = Anxiety Disorders Interview Schedule; ASI = Affective State Index; BASC = Behavior Assessment System for Children; BDI = Beck’s Depression Inventory; BDSRSC = Birleson Depression Self-Rating Scale for Children; CBCL = Child Behavior Checklist, affective problems; CDI = Child Depression Inventory; CES-D = Center for Epidemiological Studies Depression scale; CIDI = Composite International Diagnostic Interview; DASS21 = Depression, Anxiety and Stress Scale; DDPCA = Dimensions of Depression Profile for Children and Adolescents; DEQ-A = Depressive Experiences Questionnaire for Adolescents; DISC-R = Diagnostic Interview Schedule for Children Revised; DIQ = Domestic Interactive Questionnaire; DML = Depressive Mood List; DSRS = Depression Self-Rating Scale; HIF = How I Feel; HADS = Hospital Anxiety and Depression Scale; K-SADS = Schedule for Affective Disorders and Schizophrenia for School-Age Children; NPBL = Nijmegen Problem Behavior List; PANAS-C = Positive and Negative Affect Scale Children; RCADS = Revised Child Anxiety and Depression Scale; RCDS = Reynolds Child Depression Scale; SCID-I = Structured Clinical Interview for DSM-IV Axis I Disorders; TSCC = Trauma symtoms checklist for children; WAI = Weinberger Adjustment Inventory; YSR = Youth Self Report, affective problems scale
